# Native Architecture of Wheat Straw Cell Walls: A Unified Model from X‑ray Scattering and Solid-State NMR

**DOI:** 10.1021/jacs.5c23116

**Published:** 2026-03-19

**Authors:** Yucheng Hu, Pan Chen, Peng Xiao, Shixu Yu, Lingfeng Zhou, Zhe Ling, Yutong Zhu, Guohua Miao, Yuan He, Haichao Li, Sheng Chen, Tingting You, Feng Xu, Tuo Wang, Yoshiharu Nishiyama

**Affiliations:** † State Key Laboratory of Efficient Production of Forest Resources, Beijing Key Laboratory of Lignocellulosic Chemistry, 12380 Beijing Forestry University, Beijing 100083, China; ‡ School of Materials Science and Engineering, 47833 Beijing Institute of Technology, 100081 Beijing, China; § Department of Chemistry, 3078 Michigan State University, East Lansing, Michigan 48824, United States; ∥ Jiangsu Co-Innovation Center of Efficient Processing and Utilization of Forest Resources, College of Chemical Engineering, 74584 Nanjing Forestry University, Nanjing 210037, China; ⊥ Univ. Grenoble Alpes, CNRS, CERMAV, 38000 Grenoble, France

## Abstract

Plant secondary cell walls constitute the dominant reservoir of renewable biomass, comprising tightly packed cellulose, hemicellulose, and lignin at the nanoscale. Recent advances in solid-state NMR spectroscopy and the availability of small-angle X-ray scattering for biomass characterization have led to an accumulation of experimental data on cell wall organization, yet no explicit structure model has simultaneously satisfied both X-ray and NMR observations. Using wheat straw as a model system, we propose a structural framework consistent with current knowledge of cellulose biosynthesis, X-ray scattering data, and one- and two-dimensional ^13^C solid-state NMR spectra. In this model, 18-chain elementary fibrils align in parallel and populate the cross-section at random. Arabinose-substituted xylan shows no conformational dependence for cellulose-binding in wheat, and only a minor fraction of 2-fold xylan appears in close proximity to cellulose, unlike in *Arabidopsis*, where xylan is more tightly attached to the cellulose surface. While NMR data cannot unambiguously resolve the internal arrangement of the 18 glucan chains, X-ray scattering profiles uniquely constrain the fibril size and exclude the possibility of tight bundling in the intact walls. The specific interaction between the matrix polymers and the cellulose elementary fibrils must be reconsidered in light of the small interfibril spaces, which bring the matrix components into spatial proximity with cellulose even in the absence of attractive interactions. These findings provide fundamental molecular-level insight into cellulose fibril architecture and matrix–polymer interactions, resolving longstanding discrepancies between spectroscopic and scattering data and advancing our understanding of biopolymer assembly into structurally and functionally versatile lignocellulosic biomaterials.

## Graphical abstract

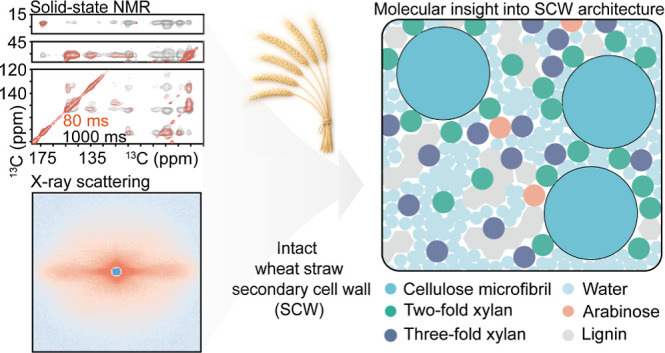

## Introduction

All land plants produce cell walls built around crystalline cellulose embedded in a water-swollen matrix of other polysaccharides and biopolymers. Primary cell walls formed by all growing plant cells consist mainly of cellulose, hemicellulose, and pectin. In contrast, many specialized cells, such as xylem vessels, fibers, and sclerenchyma, deposit thick secondary cell walls composed primarily of cellulose, hemicellulose, and lignin.^1,2^ The formation of the secondary cell wall is crucial for providing mechanical strength and resistance to collapse in dry terrestrial environments. The secondary cell wall, also termed lignocellulose, is the central component of the available biomass stock because it generally degrades slowly in nature, and its unique combination of strength, durability, workability, and wide availability has made lignocellulose a material essential throughout human history.^3–5^

The structure of cellulose is highly conserved between species, and all higher plants produce the crystalline form of cellulose I_β_ or closely related structures. Because of its simple chemical structure and crystallinity, cellulose’s structure is among the best understood. However, over many years, structural studies have focused on model systems with large crystallite sizes in purified form, yielding better-resolved data than intact cell walls of common plants. The atomic coordinates and crystal shapes of model cellulose are well established.^6,7^ The squarish cross sections with lattice images spanning the whole width have been observed for green algae, in which case the surface consists of ribbon-like cellulose chains that are obliquely exposed. Nevertheless, the shape and arrangement of cellulose in the most typical biomass remain elusive.

Cellulose is produced by the terminal complex, a set of transmembrane enzyme assemblies that move in the plane of the cell membrane and are deposited outside the membrane in crystalline form.^8,9^ The terminal complex in green algae, such as *Valonia*, for which the microfibril cross-section is established, is of a linear type, as opposed to the rosette-type in land plants. In most land plants, cellulose is synthesized in the presence of hemicellulose, which is known to prevent elementary crystals from coalescing laterally.^10,11^

For many years, each granule of the 6-membered rosettes was assumed to produce six chains simultaneously,^11,12^ forming a microfibril of 36 chains, consistent with the electron-microscopic impression of the microfibril replica, which is inherently imprecise at the nanometer scale. This situation changed with the determination of the 3D structure of a bacterial cellulose synthase,^13^ which guided the shape and size of the plant cellulose synthase, which can fit only three in a rosette granule, restraining the elementary unit to 18 chains.^14–16^

Still, larger fibrillar objects are often observed under the electron microscope, leading to the claim of the existence of macrofibrils in which several elementary fibrils bundle together.^17^ However, this model is problematic, as the massive bundling into macrofibrils contradicts the absence of electron density fluctuations at a tens-of-nanometer length scale, as evidenced by the weak X-ray scattering over a scattering vector range of 0.01 to 0.1 Å^–1^, where intensity appears only after hydrothermal treatment.^18,19^

X-ray scattering has been used for over 100 years in the study of biomass; the experimental results are very robust and easily reproducible, independent of the operator, and require only a simple setup and virtually no sample preparation, contrary to microscopic techniques.^20^ Although X-ray scattering cannot unambiguously determine the spatial arrangement of elementary fibrils, predicting an X-ray scattering pattern is straightforward given an explicit structural model.^21^

High-resolution solid-state NMR (ssNMR) is another technique used to investigate the structure of cellulose and biomass, as chemical shifts are sensitive to conformation.^22,23^ It has been recognized from the early days of its application to cellulose that the C4 and C6 resonance peaks of cellulose I crystals are shifted downfield from those in solution or in the amorphous structure.^24^ The microfibril surface is considered to have a similar conformation to amorphous, with its relatively peak intensity inversely proportional to the crystallite size.^25,26^

Hemicellulose represents an ensemble of polysaccharides produced in the Golgi apparatus and transported to the nascent cell wall in a vesicle.^27^ Xylan is one of the most common hemicelluloses, consisting of a linear β-1,4-linked xylose backbone with variable side-group substitutions, depending on plant species. In wheat xylan, arabinose is predominantly α-1,3-linked.^28^ The xylan backbone tends to form a left-handed helix,^29^ close to a 3-fold helix, but under some conditions can also adopt a flat 2-fold helical structure.^30,31^ In earlier studies of model polymers, when unsubstituted xylan adsorbs on cellulose, it forms a 2-fold helix, whereas its crystalline form in water is a 3-fold helix.^32^

Lignification of the cell wall occurs after polysaccharide deposition. Monolignols, produced in the cytoplasm, are transported to the cell wall and then polymerized by radical coupling reactions.^33,34^ These processes lead to a highly compact cell wall structure, with the three components intermixed at the nanoscale.

In recent studies of plant cell walls, ssNMR offers another advantage: it is site-specific and can probe interatomic proximities through dipolar recoupling techniques and spin diffusion mechanisms.^35,36^ The resulting distance constraints have been statistically analyzed to refine structural models and eliminate incompatible hypotheses.^37^ These models have better defined the structure-interaction relationships of molecules operating at the lignin-carbohydrate interface. In addition to 2-fold xylan bound on the cellulose surface,^38–40^ 3-fold xylan is found to be in physical contact with lignin across diverse plant species, and lignin itself serves as a secondary contact site, after xylan, for interactions with cellulose in woody stems.^41,42^ However, only limited efforts have been made to construct an explicit structural model that simultaneously satisfies both X-ray scattering and ssNMR data while taking into account the continuity of the cell wall.

In this work, we establish the framework by investigating uniformly ^13^C-enriched wheat straw (*Triticum aestivum*), for which the detailed chemical composition was reported in a previous study,^43^ using X-ray scattering and ssNMR to narrow down the potential structures of the lignified cell wall.

## Results

### Composition and Dynamics of Biopolymers in Wheat Secondary Cell Walls

1D ^13^C ssNMR spectra measured with different polarization methods, including Direct polarization (DP) combined with 2- and 40 s recycle delays, as well as cross-polarization (CP), allowed us to track the presence of functional groups and analyze their dynamics (Figure 1a). The short recycle delay in DP experiments suppresses signals from rigid molecules, whose polarization does not have sufficient time to recover to equilibrium before the next scan, thereby enhancing the relative contribution of mobile components that undergo faster spin–lattice relaxation. Because the 40 s recycle delay exceeds five times of the ^13^C-T_1_ of all molecules (3–7 s) in this uniformly ^13^C-labeled sample, the resulting ^13^C DP spectrum effectively captured all carbon atoms (>99.3%) in the system. At a 2 s recycle delay, the magnetization of rigid fragments is relaxed by only 20–40%, so the signal of the rigid fragments is reduced by roughly 30%. In the following, we consider the integration of the DP spectra from 0 to 200 ppm with a 40 s recycle delay as the total intensity, and compare the different components as fractions.

**Figure 1:**
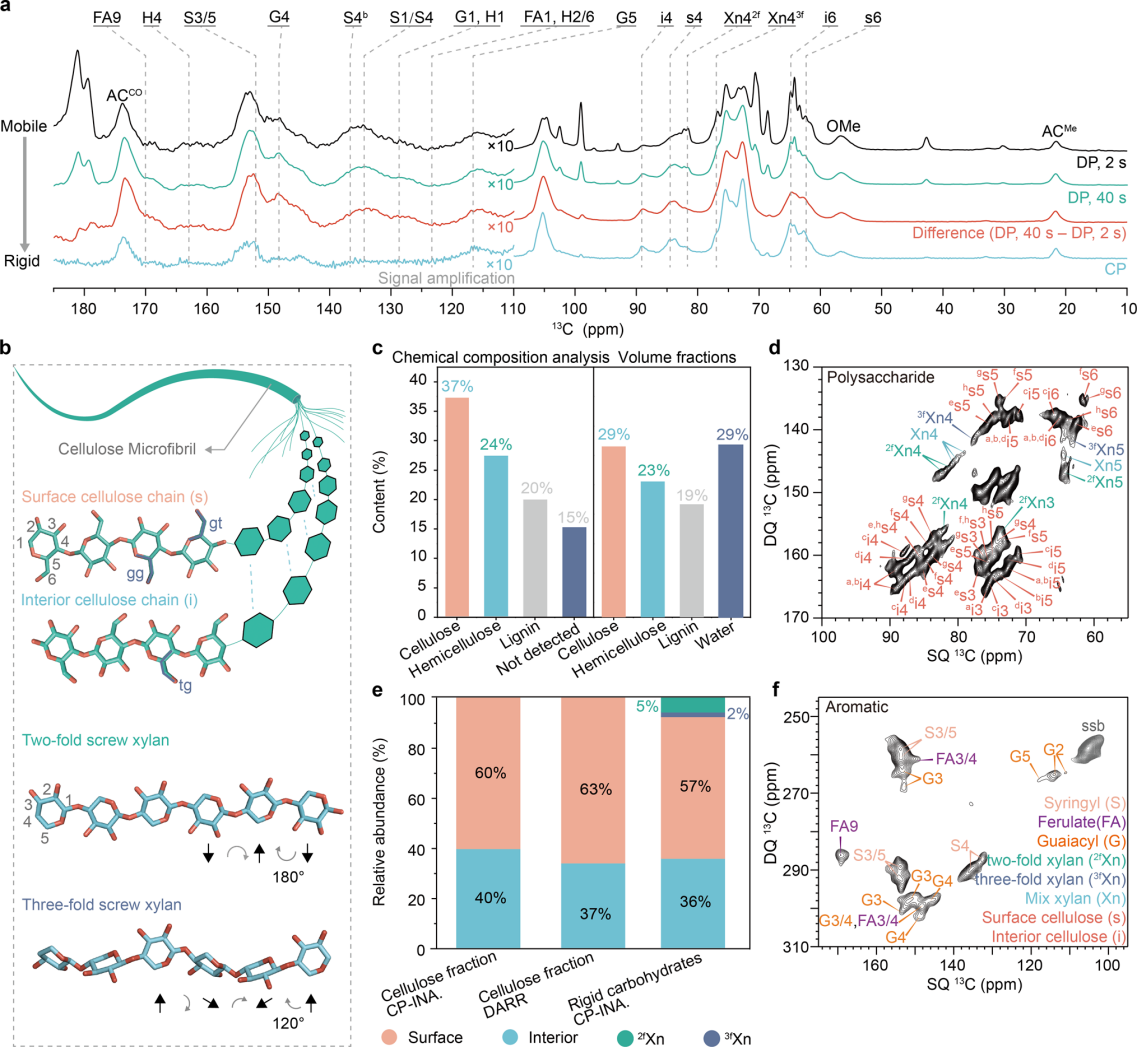
ssNMR of ^13^C enriched wheat straw at 40% moisture content. (a): 1D ^13^C ssNMR spectra. ^13^C DP with a 2 s recycle delay enhancing mobile molecule contribution, and with a 40 s recycle delay that quantitatively detects all carbons. The difference spectrum between the two DP spectra shows a relatively rigid portion of cell wall components, and the CP spectrum, which selectively detects highly rigid molecules via ^1^H–^13^C dipolar couplings. (b), Schematic description of representative structures of polysaccharides. The cellulose microfibril is composed of surface chains and interior chains with different primary alcohol conformations. The 2-fold conformation allows xylan to form a flat ribbon shape, rotating 360° every two xylose units. Xylan in the 3-fold conformation rotates 360° every three xylose units, forming a helical shape. (c), The sugar composition analysis and volume fraction calculation of the wheat straw. (d), 2D ^13^C CP J-INADEQUATE spectrum of wheat straw showing carbon connectivity in the rigid portion of polysaccharide; color-coded (^g^s5 denotes carbon 5 of glucose type-g; Xn4 denotes carbon 4 of two/3-fold mixed xylose). SSB: Spinning sideband. (e), The fraction of the interior and surface cellulose chains from the 2D CP J-INADEQUATE spectrum and the DARR spectrum; Contents of different polysaccharide components were determined from the 2D CP J-INADEQUATE spectrum (Table S2). Using chemical shift ranges for integration and peak volume determination might introduce an expected error margin of a few percent of the integrated values. (f), 2D ^13^C CP J-INADEQUATE spectrum of wheat straw showing carbon connectivity in the rigid portion of aromatic regions.

The spectral region between 60 and 110 ppm corresponds to polysaccharides, with sp3 carbons attached to oxygen, carbon, and hydrogen atoms. The peaks between 60 and 65 ppm correspond to carbon atoms attached to two hydrogen atoms, i.e., C6 of cellulose or C5 of xylan. The hydroxymethyl groups of cellulose I adopt a constrained conformation (*tg*, *trans* to O5 and *gauche* to C4) at 65 ppm (Figure 1b). In contrast, the chains on the surface, especially those with the C6 exposed to the outside of the crystal, can adopt a more relaxed conformation, mainly in the *gt* conformation.^23^ The peaks at 80–90 ppm correspond to highly constrained C4 atoms of cellulose and xylan, which adopt conformations close to a 2-fold helical structure. The C4 of relaxed conformations, close to 3-fold, appears in the upfield region buried in the overlapping peaks of C2, C3, and C5. The peak between 100 and 110 ppm is dominated by the C1 of sugars, which is the anomeric carbon with neighbors of two oxygen atoms, in the β-configuration for cellulose and xylan backbone, and in the α-conformation for arabinose side chains. The broad peaks between 110 and 170 ppm correspond to the aromatic carbons of lignin, with possible minor contributions from the double-bonded carbons in lipid acyl chains and the aromatic side chains of proteins.

The aromatic region accounted for 12% of the total, whereas the sugar region, from 60 to 110 ppm, accounted for 76%. This sugar region also contains signals from part of the propane moiety and from the aromatic C2 and C6 of lignin; thus, the intensity ratio is roughly consistent with the carbohydrate: lignin ratio of 65:20 determined by chemical analysis (Figures 2c; Note S1; S1). The 4% methoxy carbon at 55 ppm is also in good agreement with an equivalent mixture of syringyl (S) and guaiacyl (G) moieties. The cellulose-to-xylan ratio is more delicate to estimate from the 1D spectra due to the overlap of the resonance position of all carbons. However, when comparing the C1 intensity (12%) and the integration of the 80–90 ppm region that corresponds to C4 (10%), at least 83% of polysaccharides (including both cellulose and xylan) are in roughly 2-fold conformation. If we take the glucose: xylose ratio of 6:4 from the sugar component analysis to be the cellulose: xylan ratio, then 60% of xylan would be in the 2-fold conformation (7.6 2-fold xylan chains; Note S2). Both the C=O and CH_3_ carbons of the acetyl groups give a consistent value of 2%, indicating that xylan is acetylated on average every 2.7 residues.

**Figure 2:**
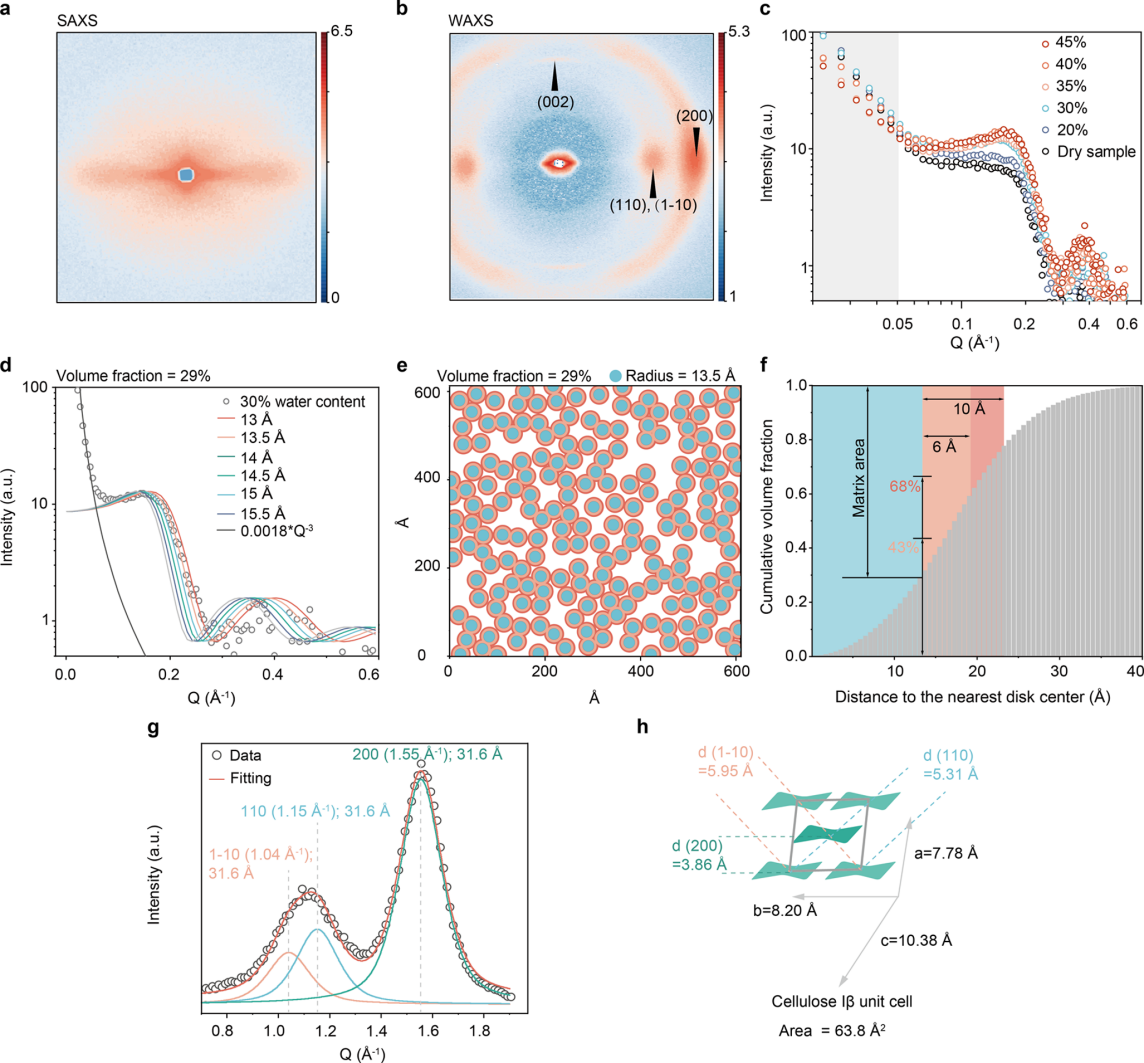
Potential architecture and arrangement of cellulose microfibrils. (a), Two-dimensional SAXS and (**b**) WAXS pattern of the wheat straw. (c), Anisotropic SAXS profiles at different water contents. (d), Comparison of experimental X-ray small-angle scattering data with simulation of randomly packed cylinders with various radius (13, 13.5, 14, 14.5, 15, 15.5 Å). The central scattering follows 0.0018*Q^–3^ and is due to the surface scattering. (e), Snapshots of randomly distributed disks at an area fraction (29%) corresponding to a physical microfibril radius of 13.5 Å at an effective microfibril volume fraction of 40%. (f), Cummulative histogram of lattice points to the nearest cylinder center. The volume fraction of the matrix within 6 Å from the surface is 43% (light pink) and within 10 Å is 68% (dark pink). (f), Regions corresponding to distances <6 Å (light pink) and <10 Å (pink) are shaded to match the matrix volumes and the NMR-derived domains. (g), Anisotropic WAXS profile extracted from the 2D pattern and fitted with pseudo-Voigt functions. (h), Unit cell of cellulose Iβ from tunicate (Table S4).^7^

### Intensity Analysis of the C4 Region in 2D ssNMR Spectra

The signals of rigid polysaccharides, heavily overlapping in the 1D spectra, can be better separated in the 2D ^13^C refocused J-INADEQUATE spectrum (Figure 1d, Table S1). Especially, the C4 signals from cellulose and from xylan are well separated in this spectrum, due to the substantial difference in the double-quantum (DQ; vertical dimension) chemical shift of the C4–C5 carbon pairs in these two polymers. The DQ chemical shift corresponds to the sum of two single-quantum chemical shifts of a directly bonded carbon pair. The difference arises largely because C5 is an exocyclic carbon in xylan, whereas it is not in cellulose. In addition, the difference in the C4 single quantum chemical shifts of the C4 sites in xylan and cellulose also contribute to the observed DQ chemical shift separation of the C4–C5 pair. As such, xylan C4 peaks vertically shifted by 15–20 ppm in its DQ chemical shift from the cellulose C4 clusters. The 2-fold xylan peak (^2f^Xn4) has a long tail toward the 3-fold xylan peak (^3f^Xn4) at 78 ppm with relatively continuous conformation distribution (Xn4 in between).

The clustered peaks can be separated into eight types of glucose residues: embedded in the core (^c^i), those underneath the surface (^a^i, ^b^i, and ^e^i), and those preferentially residing on the surfaces (^e^s, ^f^s, ^g^s, and ^h^s)^23,44^ (Figure 1d). Their signals are heavily overlapped, so to estimate their composition, we will treat them only as “interior”, i.e. downfield (>86 ppm) and “surface”, i.e. upfield (<86 ppm) groups. We refer to these signals as “interior” and “surface”, since the upfield component is, on average, closer to water and surface-bound molecules;^23,45^ however, ongoing efforts are underway to more precisely determine their molecular identities.^46^

Similarly, xylan conformation can be categorized into two groups: 2-fold xylan (>80 ppm, ^2f^Xn) and 3-fold (<80 ppm, ^3f^Xn), keeping in mind that there is a continuum of signals in between resulting from conformational distribution or acetylation complexity. Within the rigid fraction, around 5% are ^2f^Xn and 2% are ^3f^Xn (Figure 1e and Table S2). The 3-*O*-arabinose and 2-*O*-linked glucuronic acid substitutions of xylan chains were mostly observed in the mobile phase due to the flexibility of these side chains (Figure S2 and Table S3). 2D ssNMR and solution-state HSQC spectra revealed signals from S, G, ferulate (FA), and *p*-hydroxyphenyl (H) units within the aromatic domain (Figures 1f and S3). Solution-state HSQC does not capture the whole cell–wall composition because it probes only the solubilized fraction of the sample, however, it provides better resolution of the chemical diversity of biopolymers.

The molar fractions of different conformers among the rigid cellulose and xylan from the integrated peak area are summarized in Figure 1d. Joint analysis of CP-INADEQUATE and dipolar-assisted rotational resonance (DARR) spectra constrains the “interior” cellulose chain fraction to 37%-40% (Figures S4–S6 **and** Table S2).

The interior cellulose chain fraction is an operational definition based on chemical shift and dynamic environments, and is not strictly equivalent to a spatial position. Among different possibilities of cross-sectional arrangement for an 18-chain microfibril (Figure S6), the number of interior cellulose chains always falls between ∼ 6.6 and 7.2, which covers the experimentally derived interior-chain fraction (Notes S1 and S2).^47,48^

### The Size and Arrangement of Cellulose Fibrils

Figure 2a,b show the X-ray small- and wide-angle scattering image, respectively, of straw with a moisture content of 35% with the vertical axis parallel to the stem. The horizontal streak in the small-angle pattern reflects the electron density fluctuation in the cross-section of highly oriented fiber cells at a nanometer length scale. Some broad components form an average angle of 40° with the horizontal, reflecting the helical structure of the parenchyma cell’s secondary cell wall. The wide-angle pattern (Figure 2b), corresponding to fluctuations at subnanometer length scales, shows two diffraction spots along the horizontal line (azimuthal) corresponding to the 1–10/110 composite peak and the 200 reflections. The same azimuthal-angle dependence as in the small-angle streak is seen in the intensity variation along the azimuthal line of the 200 peak, indicating the same texture at both length scales. The sharpness of the diffraction broadening in the radial direction directly reflects the extent of regular structure, and a sharp 002 diffraction spot in the vertical (meridian) direction shows the regularity of the structure along the chain direction, as compared to the broad equatorial diffractions and thus small lateral dimensions of the crystal.

The intensity of the small-angle equatorial streak was separated from the isotropic scattering by fitting according to the previously developed approach^19^ and shown as a function of the scattering vector *Q* = 4πsin­(θ)/λ with Lorentz correction (multiply by Q), in a double logarithmic plot (Figure 2c) for samples with different water content. The small-angle regime below 0.05 Å^–1^ is dominated by surface scattering, typically at the boundary between the lumem and the cell wall, and follows a Q^–3^ behavior. The relatively low intensity between 0.05 and 0.1 Å^–1^ clearly indicates the absence of macrofibrils with diameters of 5–10 nm. The intensity below *Q* = 0.5 Å^–1^ starts to decrease above 35% water content, whereas the intensity at around *Q* = 0.2 Å^–1^ increases with water content up to 30%. The 30% is a typical fiber saturation point of lignified cell walls that a cell wall can host, above which the water is condensed out of the cell wall.^49^ The intense scattering below *Q* = 0.05 Å^–1^ is dominated by interface scattering between the lumen (air) and the cell wall substance with a high electron density contrast, which decreases as a part of the lumen is filled with liquid water. The intensity at around *Q* = 0.2 Å^–1^ is considered a correlation peak of the cellulose microfibrils, and arises from the contrast between cellulose and the matrix component. Swelling of the matrix component by water increases the electron density contrast relative to the dry state, resulting in increased intensity up to 30%, again confirming the fiber saturation point.

As a first approximation, we consider the cell wall to be composed of two phases: (1) cellulose microfibrils and (2) a matrix component comprising hemicellulose, lignin, and water. The equatorial scattering intensity profile depends on the form factor of cellulose, which we assume to be cylindrical, and on its lateral disposition that determines the structure factor. Since we have an estimate of the cellulose volume fraction, a random packing yields a unique structure factor. We calculated a series of scattering profiles at a volume fraction of 29% and varying cylinder radii to assess their effect on the scattering profile (Figure S7). The shoulder position shifts with the cylinder radius, with the larger radius showing the shoulder at a smaller angle. However, the profile does not reproduce the decrease in intensity toward lower angles, which is predicted only at higher volume fractions. Assuming a sheath layer that prevents the cylinder from approaching closely, so that the effective volume fraction is 40%, led to an almost perfect match to the experimental curve when the cylinder radius was 13.5 Å. This situation is illustrated in Figure 2d, where cylinders are randomly packed with a minimum separation of 4.7 Å, with the zone near the cellulose colored in red. Among the whole matrix volume, 43% is within 6 Å distance to the cellulose surface, and 68% is within 10 Å distance (Figure 2e,f).

The diffraction profile along the equatorial line shows only two peaks, corresponding to an orthorhombic unit cell, classically called cellulose IV.^50,51^ However, it can also be interpreted as due to the broadening of the diffraction from the monoclinic unit cell, with two separate peaks constituting the 110/1–10 composite peak. Fitting pseudo-Voigt peak functions to the equatorial diffraction profile extracted from the 2D data gives a Scherrer size of about 31.6 Å, and the peak position of 200 corresponds to a *d*-spacing of 4.05 Å. In the unit cell of the model system, d_200_ is 3.86 Å, so the microfibril unit cell is swollen in *a* direction by about 5%. This larger *d*-spacing is commonly observed in thin crystallites. Three factors contribute to this large *d*-spacing: (1) the smaller attractive London interaction, which is purely additive and operates as external pressure;^52^ (2) the potential contribution of the matrix molecules that are at slightly larger distances than the tight packing of 4 Å;^21,53^ and (3) the purely mathematical effect of the limited size.^50,51^ Overall, the unit molecular packing of the microfibril can be looser than the ideal crystal, with a cross-sectional area larger by up to 5%. The larger estimated Scherrer size than the expected microfibril size has also been reported, but can arise from the choice of peak functions and the constructive contribution from neighboring matrix molecules, as shown by the effect of the hydration layer in the X-ray scattering simulation.

To assess the involvement of xylan in fibril structure and refine the interfibril arrangement, we measured a 2D PDSD spectrum with a long mixing time (1 s) to probe intermolecular interactions and compared it with the short mixing (80 ms) DARR spectrum, which only exhibits intramolecular interactions (Figure 3a). The additional cross-peaks that are exclusively shown in the PDSD spectrum can pinpoint the exact interacting carbon sites between different carbohydrate polymers. Strong interchain cross-peaks s6-i4, i6-s4, and s4-i6 (Figure 3b,c) were observed between the internal and external cellulose chains, and these carbon sites are separated by approximately 6 Å. However, we did not observe the expected xylan-cellulose cross-peaks, such as s6-^2f^Xn4, i6-^2f^Xn4, ^2f^Xn4-i6, ^2f^Xn4-s6, and s4-^2f^Xn5, which remain ambiguous or absent in the spectrum of wheat (Figure 3b,c, dashed boxes). This observation suggests that most 2-fold xylan chains are either positioned further away from the microfibril surface than the typical interchain distances within a cellulose microfibril, or that only a small fraction of the 2-fold xylan population remains in contact with cellulose. Consequently, they do not form extensive or close adhesive contacts, aligning with our SAXS results in which no xylan contributes to the apparent cross-sectional scattering signal (Figure 2c). When microfibrils are randomly packed, ∼ 43% of the matrix phase is located within 6 Å of the microfibril (Figure 2e,f). To reconcile this potential near-surface volume with the scarcity of matrix-cellulose contacts, we hypothesize that the rigid matrix polymers are unfavored within 6 Å shell, while a small fraction of chains—particularly 2-fold xylan segments—may sporadically approach this regime (Figure 2e,f). Therefore, the “non-penetrating interfacial layer” on the cellulose microfibril surface was introduced to keep interfibrils at a finite separation consistent with the SAXS-derived cross-sectional constraints. Since cellulose microfibrils are quite rigid, even a small molecule inserted between two microfibrils would impede their close approach. We also note that the pattern we observed here in wheat differs from what has been reported in *Arabidopsis* in which cross-peaks between 2-fold xylan and both interior and surface cellulose were clearly identified at similar measurement conditions (Figure 3c–e).^14^ This discrepancy presents another evidence that the idea that carbohydrate composition and cell–wall organization are species-specific.^42^ Indeed, when comparing with other commelinid monocots (e.g., maize, or *Zea mays*) and the dicot plant and hardwood model *Arabidopsis*, the acetyl groups in wheat showed weak interactions with only some population of surface cellulose whereas extensive interactions with both interior and surface cellulose are observed in maize and *Arabidopsis* (Figure 3d). This additional evidence signifies a much looser carbohydrate configuration in which the rigid xylan domains are mostly excluded from the microfibril surface and only sparsely exist at an approximately 6 Å boundary or beyond (Figure 2e,f), as opposed to the much tighter xylan-cellulose binding in maize and *Arabidopsis*, further emphasizing the diverse carbohydrate organizations across different plant species.

**Figure 3:**
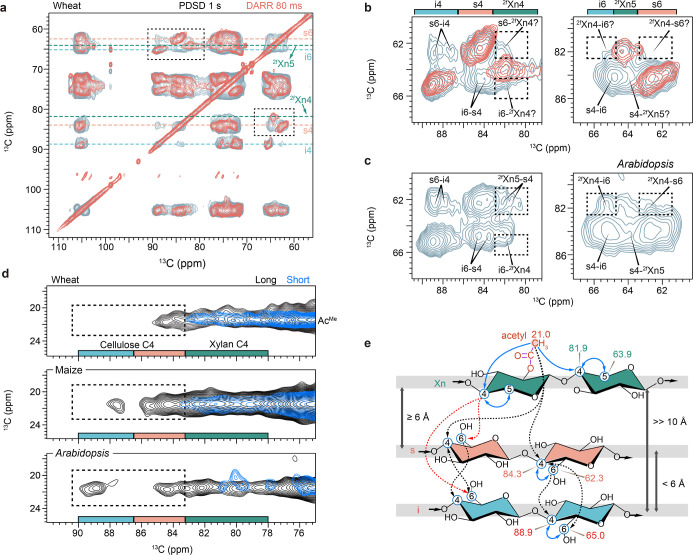
Hemicellulose-cellulose contacts probed by ssNMR. (a), Overlay of two 2D ^13^C–^13^C spectra showing short-range (80 ms DARR, orange) and long-range (1.0 s PDSD, pale blue) correlations observed in wheat straw. Key carbon sites are highlighted by dashed lines for i, s, and ^2f^Xn. (b), zoomed view of the black dashed boxed regions in panel a showing C6/C5–C4 (left) and C6/C5–C4 (right) correlations. The chemical shift ranges of key carbon sites in the F_2_ dimension are indicated as color-coded bands at the top of the panel. Only the signature cross peaks expected for intermolecular interactions are labeled. The cross peaks in **b**, wheat straw are compared with the same spectral region in (c), *Arabidopsis*. The evidence of xylan-cellulose cross-peaks is highlighted by black dashed boxes. (d), Cross peaks between acetyl methyl group (Ac^Me^) and carbohydrate C4 for wheat (top), maize (middle), and *Arabidopsis* (bottom). Short-range (Gate-DARR, 80–100 ms) and long-range (Gate-PDSD, 1.0 s) correlation spectra are shown in blue and black, respectively. The cross-peaks between Ac^Me^ and Cellulose C4 are highlighted in the dashed boxes. (e), Structural representation of the cross-peaks observed in the wheat. Intramolecular cross-peaks are shown as blue solid lines, unambiguous cross-peaks are black dashed lines and ambiguous/weak peaks are red dashed lines. Gray bands represent the position of each carbohydrate chain, and estimated ranges of interchain distance are given in between. Panel **c** is adapted with permission from ref 38 (open access). Copyright 2016 Springer Nature. The spectra of maize and *Arabidopsis* in Panel **d** were replotted with permission from ref 41 (open access). Copyright 2019 Springer Nature.

### Dynamics and Hydration Profiles of Biomolecules in the Heterogeneous Cell Walls

To rationalize the location of fibrils and associated molecules within the native, heterogeneous cell wall, we measured the dynamics and hydration of biomolecules using four ssNMR techniques. The dipolar order parameter (S_CH_) was determined using a dipolar-chemical-shift correlation (DIPSHIFT) experiment.^54^ This experimental scheme was carried out using two polarization approaches: CP for rigid molecules and 40 s DP for quantitative analysis of all molecules (Figure S8). Cellulose is intrinsically rigid, displaying large, near-unity S_CH_ values for i4 and s4 sites regardless of polarization methods, while xylan exhibits a broad range of dynamics, with large S_CH_ values of 0.90–0.95 in the rigid domain associated with cellulose and 0.7–0.8 for all xylans within the cell wall (Figure 4a and Table S5). This provides evidence of the dual function of xylan in both interacting with cellulose microfibrils, thereby becoming rigidified, and in forming the mobile matrix.

**Figure 4:**
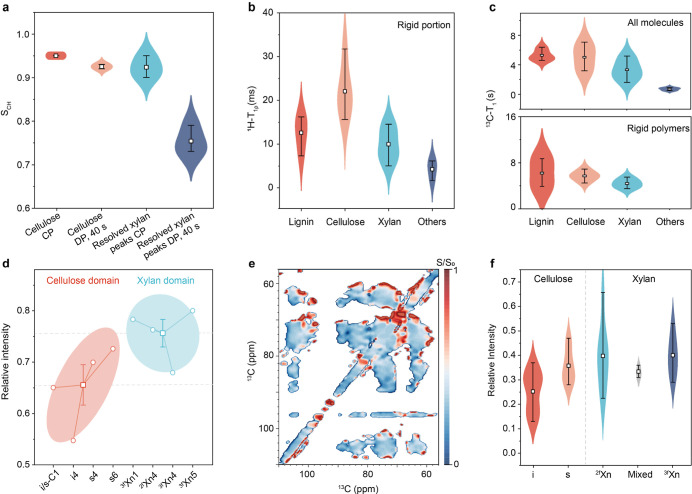
Carbon-specific hydration and dynamic landscape of polysaccharides and lignin. (a), ^13^C–^1^H dipolar order parameters (S_CH_, 0 < S_CH_ < 1) of cell wall polysaccharides. *n* = 2, 2, 3, and 3 for CP cellulose (i4 + s4), DP 40 s cellulose (i4 + s4), CP resolved xylan peaks and DP 40 s resolved xylan peaks, respectively. The open box represents the mean and the error bars are s.d. (b), ^1^H-T_1ρ_ relaxation times of cell wall polymers (*n* = 4, 4, 4, and 3 for lignin, cellulose, xylan, and other matrix carbohydrates, respectively; mean and s.d.). (c), ^13^C-T_1_ relaxation times of all molecules (top panel; *n* = 6, 5, 5, and 5) through quantitative DP detection, and rigid molecules (bottom panel; *n* = 3, 5, and 5 for lignin, cellulose, and xylan, respectively) as indicators of nanosecond-time scale motions. (d), Water ^1^H spin diffusion in cellulose and xylan, demonstrating the interaction of water with polysaccharides (mean and s.d.). (e), Hydration intensity ratio (*S*/*S*_0_) plotted as a 2D spectral heat map in the polysaccharide region, where S and S_0_ correspond to the water-edited and the control spectra, respectively. (f), The distribution of relative water-edited intensities (*S*/*S*_0_) of different carbohydrate and lignin forms and sites: *n* = 12, 8, 7, 3, and 6 for i, s, ^2f^Xn, mixed, and ^3f^Xn, respectively. The open box represents the mean and the error bars are s.d.

Because the T_1_ and T_1ρ_ relaxation times measured here probe the spectral density of molecular motions at hundreds of MHz (nanosecond time scale) and tens of kHz (microsecond time scale), respectively, their absolute values do not uniquely determine correlation times. Accordingly, we use these relaxation times as comparative indicators of molecular dynamics under identical experimental conditions. Recent studies of secondary plant cell walls consistently report shorter T_1_ and T_1ρ_ relaxation times for components exhibiting more pronounced molecular motions.^55–58^ In addition, solid-state NMR studies of plant cell–wall materials commonly observe longer ^13^C-T_1_ relaxation times at higher magnetic fields, indicating that these motions fall within the slow-motion regime.^59,60^ In this regime, enhanced molecular mobility leads to faster relaxation, such that shorter relaxation time constants reflect more pronounced dynamics on the corresponding time scales.

Consistently, xylan and other carbohydrates in the matrix had short ^1^H-T_1ρ_ relaxation time constants of <15 ms (Figures 4b and S9), reflecting more pronounced motions on the microsecond time scale. In contrast, the cellulose domain exhibits the longest ^1^H-T_1ρ_ relaxation times (15–30 ms), consistent with reduced dynamics at this time scale due to conformational constraints within the microfibrils. The ^1^H-T_1ρ_ time constants of the surface sites are 5–10 ms shorter than those of the interior sites (Table S6), indicating that enhanced microsecond dynamics for the surface chains relative to the interior chains.^61–63^ The ^1^H-T_1ρ_ time constants of lignin fall between those of cellulose and matrix polysaccharides, revealing its semidynamic nature on the microsecond time scale (Figure 4b).

With quantitative DP detection of all molecules, the ^13^C-T_1_ relaxation time constants decreased in the order of lignin, cellulose, xylan, and other matrix carbohydrate components (Figure 4c and Table S7). The relatively short ^13^C-T_1_ relaxation time of xylan and other matrix components indicates more pronounced motions at nanosecond time scale compared to cellulose and lignin. The long ^13^C-T_1_ relaxation time of lignin and cellulose carbon sites reveal limited motions of these biomolecules in the secondary cell walls at this time scale, consistent with previous findings in other grass species, such as *Z. mays*.^41^ A similar trend was observed for the rigid molecules detected using CP, despite the loss of signals from the highly mobile components in the matrix. Notably, the ^13^C-T_1_ relaxation of lignin in the rigid fraction was highly heterogeneous, probably due to the spin-exchange between a portion of lignin and polysaccharides in a close proximity,^37,42^ while other lignin polymers remain within hydrophobic nanodomains.^41,64^

The hydration profiles of biomolecules were examined through water-editing experiments,^65,66^ which showed preferential hydration of xylan over cellulose (Figures 4d and S10). The hydration profiles were quantified by measuring the water-edited intensities (*S*/*S*_0_) of 31 carbon sites from various carbohydrates and lignin units, which were resolvable in a 2D hydration heatmap (Figures 4e, S11, and Table S8). The retained intensity of the 2-fold xylan in the water-edited spectra suggests that these structures have relatively strong interactions with water molecules. This observation reinforces the limited interactions between 2-fold xylan and cellulose and explains why 2-fold xylan can retain a high hydration level similar to its 3-fold counterpart (Figure 4f). Among all polysaccharides, the interior cellulose exhibited the lowest average hydration levels, followed by xylan of the intermediate conformation (Figure 4f). The former is expected, as the core of each microfibril consists of relatively inaccessible cellulose, while the latter is unexpected and may suggest that the intermediate conformations between the 2-fold and 3-fold are caused by the entrapment in partially dehydrated domains, which could also account for the broad conformational distribution. It corroborates the fact that the intermediate conformations of xylan were most pronounced in fully dehydrated *Arabidopsis* and some wood stems,^42,57^ but were not observed in well-hydrated maize, rice, and switchgrass stems.^41^ A slightly higher hydration level was observed for the surface chains of cellulose and the 2-fold xylan chains. Together with SAXS, PDSD evidence indicates proximity, but not tight contact, between the 2-fold xylan and the cellulose surface.

### The Patterns of Biomolecule Packing in the Cell Wall

To investigate polymer packing in lignified wheat cell walls, we measured 2D dipolar-gated DARR (80 ms) and PDSD (1 s), the latter of which yielded 97 intermolecular cross-peaks on the subnanometer scale (Figure 5a). For example, magnetization transfers from the acetyl methyl of xylan (Ac^Me^; source) to site 3 or 5 of lignin (S3/5; sink) resulted in a cross peak at (21, 153) ppm. These intermolecular interactions are classified into five types based on the structural motifs of the contacting polymers: (i) cellulose and xylan acetyl groups, (ii) lignin and cellulose, (iii) different lignin units, (iv) lignin and xylan, and (v) lignin and unresolved carbo (Figure 5b,c and Table S9). Notably, the cross-peak between the acetyl methyl group and carbon 4 of internal cellulose chains (AcMe-i4) is also absent, in contrast to the significantly stronger cross-peak observed in other plant species, further confirming the limited contacts between xylan and cellulose in wheat.

**Figure 5:**
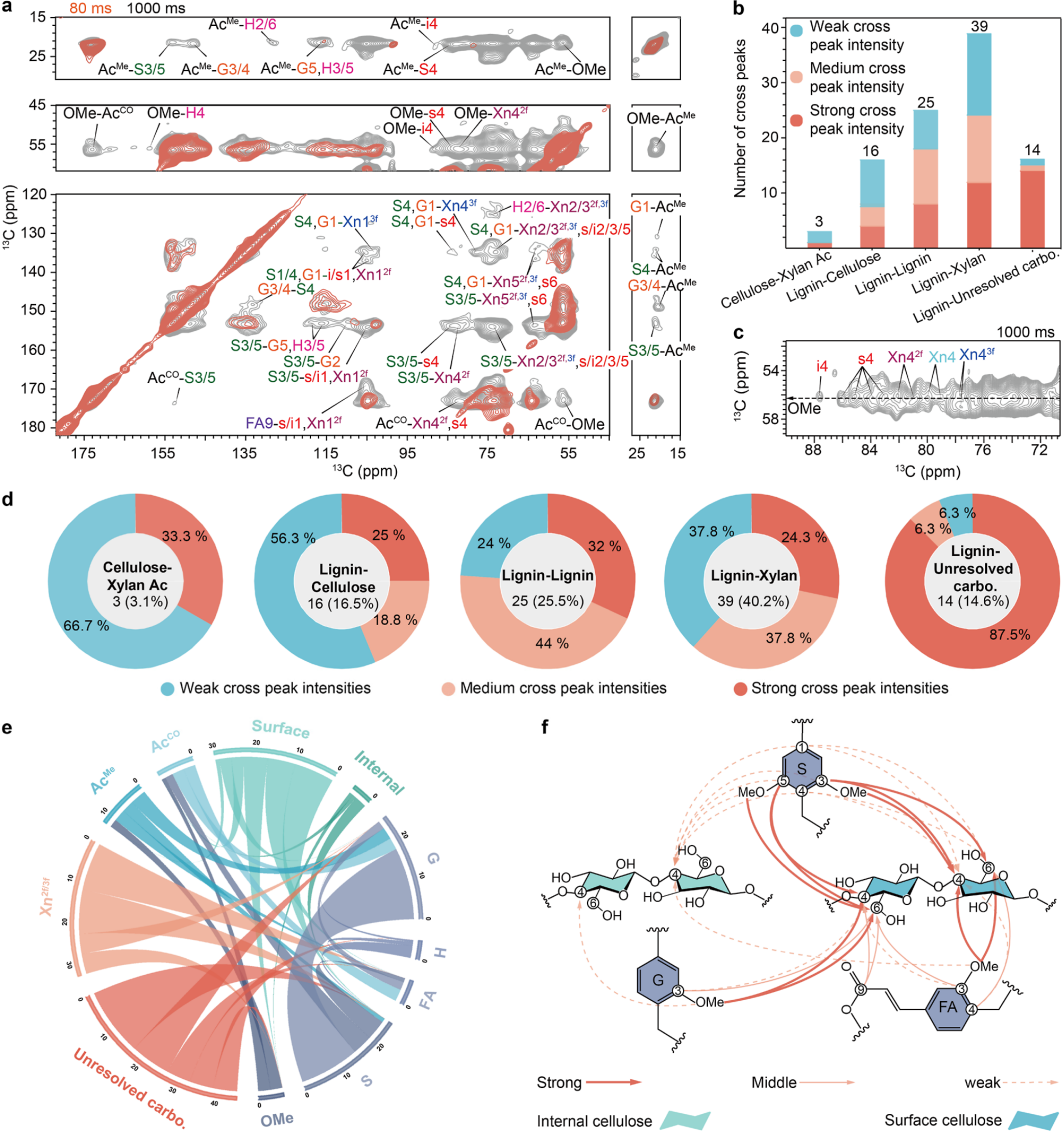
Detection of intermolecular interaction pinpointing packing interactions between polymers. (a), 2D ^13^C correlation spectra with DARR (80 ms) and PDSD (1 s) mixing. Intermolecular interactions are identified by the cross-peaks that only show up in the PDSD spectrum. Ac^Me^: acetyl methyl; Ac^Me^: acetyl carbonyl; OMe: methoxy group. Cross-peaks are labeled as “source-to-sink”, e.g., Ac^Me^-S3/5 denotes the source Ac^Me^ to sink S3/5. (b), Summary of cross peaks. In 1D slices extracted from 2D gated PDSD spectra, peaks with intensities greater than 4% are classified as strong (dark orange), those between 2% and 4% are considered medium (light orange), and peaks with intensities below 2% are categorized as weak (light blue). (c), Zoomed view of the region showing cross peaks between lignin-OMe and cell wall polysaccharides from dipolar-gated 1 s PDSD spectrum. (d), The percentage of strong, medium, and weak cross peaks within each type (e.g., strong peaks account for 33% in cellulose-xylan acetyl cross peaks). (e), The cross-peaks among molecules in the wheat straw were visualized via chord diagrams. Each arc represents a source or sink, while the lines connecting the circles illustrate the interactions between the two components. The thickness of the line represents the strength of cross peaks. (f), Structural summary of lignin-cellulose packing interactions. Arrows show the direction of polarization transfer from source to sink; Thick lines represent strong intensity, thin lines medium intensity, and dashed lines weak intensity.

These intermolecular cross-peaks are further classified as strong, medium, and weak. The most extensive interactions were observed between lignin and xylan, with 39 cross peaks (Figures 5b, S12 and Table S9), 62% of which are either strong or medium cross peaks (Figure 5d). This supports xylan’s predominant role in initially anchoring and subsequently packing with lignin in the secondary cell wall. More interestingly, lignin, particularly its methoxyl (OMe) units, exhibited equal spatial proximity to 2-fold than 3-fold xylan (Figures 5c and S12a), suggesting that distinct xylan conformers mediate different functional roles in lignin. In line with recent computational studies, some 2-fold flat-ribbon xylan chains orient their acetyl groups toward lignin when adsorbed onto the cellulose surface.^37^ The next most dominant interactions occur between different monolignol units, with 25 cross-peaks observed, 76% of which are strong. This is expected, as these units are covalently linked to form a three-dimensional heterogeneous, amorphous, and hydrophobic polymer network.

Lignin also exhibited correlations with cellulose as a secondary interaction site, with 16 cross-peaks observed (Figure 5d). However, most (56%) of these cross-peaks were very weak, indicating that the average spatial proximity of cellulose–lignin interactions is lower than that of cellulose-xylan (Figure 5c,d). This is further evidenced by the relatively weak cross peak between lignin OMe and surface cellulose, and by the almost negligible cross peak with interior cellulose (Figure 5c), the latter of which is either at the limit of 1 s ^13^C spin diffusion reach (approximately a nanometer) or present in only a very minor population.^67^ These observations align with recent findings that cellulose–lignin interactions typically occur in crowded systems, particularly in mechanically strong woody stems.^42^

The quantity and strength of intermolecular cross-peaks highlight the critical role of carbonyl and methyl carbons in acetyl groups (Ac^CO^ and Ac^Me^) in stabilizing xylan’s interactions with other molecules (Figure 5e). These key carbon sites exhibited strong interactions with methoxy (OMe) and ring carbons of S and G units of lignin, as well as the surface chains of cellulose microfibrils (Figure 5f). S units also showed multiple correlations with surface cellulose, while G units did not, indicating that the additional methoxy group increased the tendency of establishing physical contact between the lignin and the fibrillar surface.^68^

## Discussion

We propose a schematic illustration of the secondary cell wall that integrates the molecular sizes and our current data (Figure 6). This illustration respects the component volume fractions determined by chemical analysis and the known component densities, as well as their approximate sizes.

**Figure 6:**
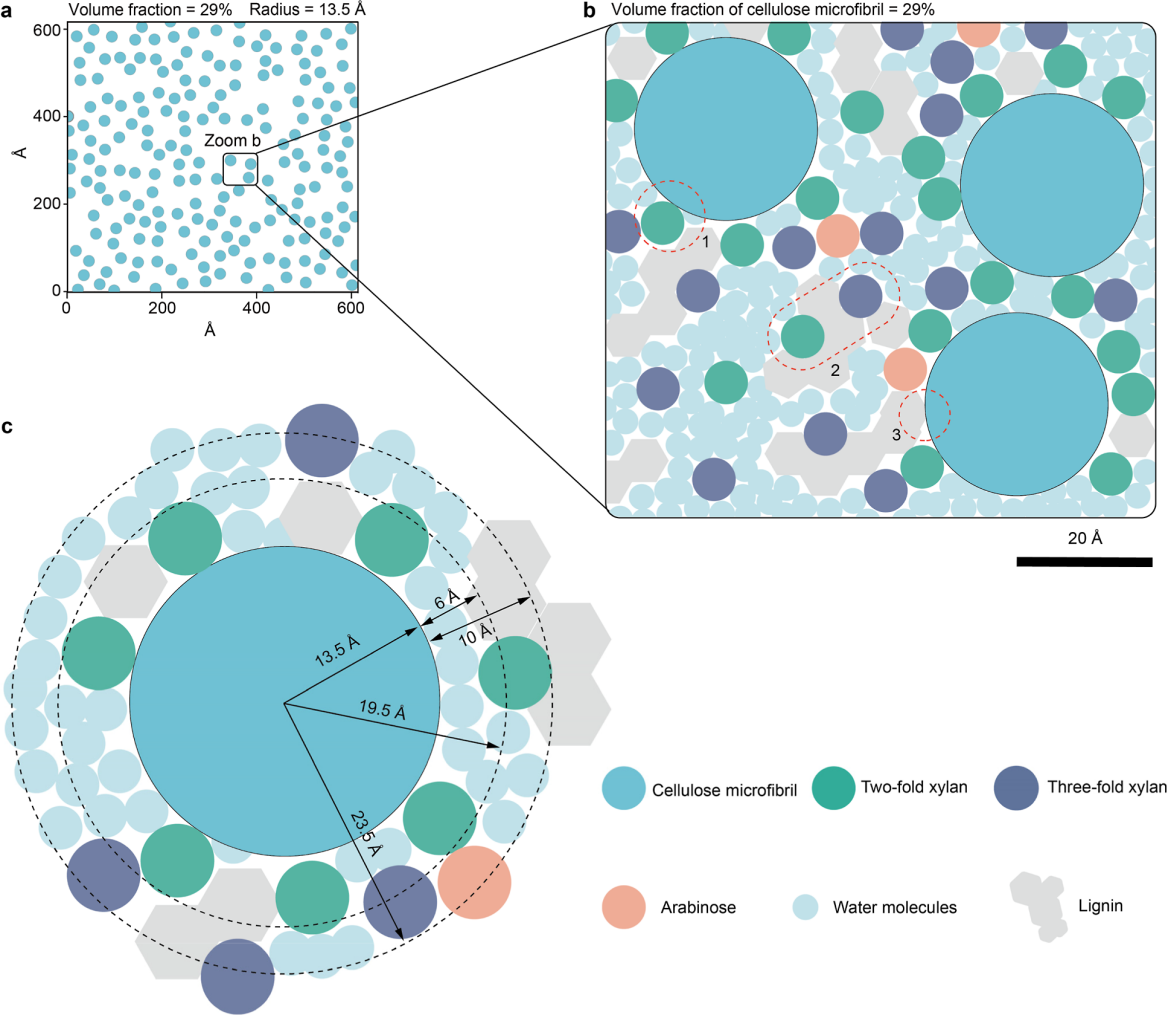
Conceptual diagram of cellulose microfibrils in the cell walls of wheat straws. (a), Snapshots of Metropolis Monte Carlo–generated randomly distributed disks at an area fraction (29%) corresponding to a physical microfibril radius of 13.5 Å at an effective microfibril volume fraction of 40%. (b), A local zoom-in view of the Monte Carlo–generated packing in (a), illustrating the random cross-sectional arrangement of cellulose microfibrils. The matrix components is crowded in the interfibrils constrained by X-ray and ssNMR. The simulation details are provided in Note S4. (c), Proximity constraints for cellulose microfibril within 6 Å and 10 Å from ssNMR. This diagram presents the spatial organization of lignin, cellulose, 2-fold xylan, 3-fold xylan, and water molecules in wheat straw’s cell wall and is plotted according to volume fraction. The numbered regions circled by dashed lines highlight specific features on the polymer interaction interface: (1) the 2-fold xylan can bind with cellulose surface behaving as matrix-like molecules; (2) the lignin domain was in contact with the cellulose surface; (3) the lignin domain was in contact with the 2-fold and 3-fold xylan. Explicitly state that the final schematic represents a quantitatively constrained structural model, rigorously bounded by SAXS, WAXS, ssNMR, and sugar analysis. The polymer composition ratios used in the model are detailed in Figure S13.

Cellulose aggregated into 18-chain microfibrils with an equivalent diameter of 13.5 Å (density of 1.6 g/cm^3^) occupying a 29% volume fraction. The microfibrils are noncontacting, with a minimum surface separation of 4.7 Å, corresponding to an effective radius of 15.9 Å of nonpenetrating cylinders (Figures 2d–f and 3, 6a). This separation corresponds to the thickness of a polysaccharide or lignin fragment that can be inserted anywhere between two rigid cellulose microfibrils as a wedge to impede their further approach. We assume there is no attraction or repulsion between microfibrils, which leads to a random distribution of the disk in the cross-section.

The number of xylose and arabinose residues and lignin monomers per cellulose microfibril is determined from the sugar composition analysis (glucose: xylose: arabinose = 53:37:4), yielding about 12 xylose and 1 arabinose per microfibril. The carbohydrate-to-lignin mass ratio from the chemical analysis is 65:20. Assuming a moisture content of 30% on a dry-base basis, for every 18 glucose residues, there are 12 xylose residues, 1 arabinose residue, 7 lignin residues, and 110 water molecules. The ratios of xylose and arabinose to cellulose could not be independently confirmed by NMR due to severe signal overlap in the 1D spectra and the limited quantitative accuracy of 2D NMR. The 2D NMR spectra show much stronger cellulose signals than hemicellulose signals, but this may reflect differences in their dynamic behavior. The volume occupied by the partially acetylated xylose residue is roughly equivalent to that of glucose and thus drawn with a 3 Å radius. The water molecule has an effective radius of 1.9 Å and occupies 30 Å^3^. Accounting for the linear density along the microfibril direction, which is lower for water than for the polymer, we present 60 water molecules per microfibril, each with a radius of 1.9 Å, colored in light blue.

The population of two-to 3-fold xylan is taken from 2D NMR spectral integration and is in good agreement with the total conformation ratio of beta-1,4-linked pyranose estimated from the C4 region of quantitative NMR (40s DP NMR). So among the 12 xylose, 7 are in a 2-fold conformation, colored in green, and 5 in a 3-fold conformation, colored in dark blue (Figure 6b). Arabinose is always linked to xylan, which can be 2-fold or 3-fold.

In wheat, arabinose-substituted xylan shows no conformational selectivity for cellulose association; only a minor fraction of 2-fold xylan is observed in proximity to cellulose (Figure 6b region 1). Thus, 2-fold xylan can behave primarily as a matrix component near, but not adhered to, the fibril surface (>6 Å). Interestingly, 2-fold xylan and 3-fold xylan exhibit comparable proximity to lignin (Figure 6b region 2). Direct lignin-cellulose contacts are also detected but occur less frequently than lignin-xylan contacts (Figure 6b region 3). These packing relationships, particularly matrix-cellulose proximity, were constrained by the ssNMR-detectable distances (6 Å and 10 Å; Figure 6c). When xylan does not tightly adsorb on the microfibril surface, it occurs mainly at ∼ 6 Å or beyond when randomly occupying the interstices of microfibrils. Collectively, the model suggests that the small interfibril spaces can bring matrix polymers into spatial proximity with cellulose even in the absence of attractive interactions. These are prompting a reassessment of the specific interaction between the matrix polymers and the cellulose microfibrils.

Emerging insights from scattering techniques, ssNMR, and modeling of the cellulose synthase complex,^16,69^ along with cellulose microfibrils synthesized in vitro and located in muro,^70,71^ increasingly converge to support an 18-chain elementary microfibril model.^72^ Some of the previous ssNMR and X-ray diffraction studies have sometimes favored a canonical 24-chain fibril model or binding arrangement,^62,63^ but those models were built on unbased assumption that all surface molecule takes the same conformation, or a wrong assumption of detecting scattering from an isolated microfibril. Our data show that the cellulose microfibrils in wheat straw remain randomly arranged under hydration. The existence of a large macrofibril is more frequently reported in woody plants. However, the macrofibril models are often based on images after aggressive sample preparation or signal filtering^17^ with a high propensity for artifacts.

The “surface” to “interior” signal ratio of ssNMR is often used to discriminate among different possible arrangements of chains in a microfibril (Figure S6). However, the C6 hydroxymethyl groups of the surface chains can point toward the fibril interior every other residue, and can contribute to the interior cellulose chain population. In this study, due to the severe overlapping with the C5 of xylan, we did not quantify the C6, but the C4 chemical shift can also be correlated with the C6 conformation, leading to the effective number of interior chains being challenging to determine. The upper bound corresponds to the conventional interpretation that all residues of the surface show the “surface” signal. The lower bound assumes that some outer chains, whose C6 groups point toward the fibril interior, contribute to the interior-like population. The often drawn 234432 arrangements (a and b) indeed are close to the upper bound, and quantum mechanical energy calculation is in support of such a model. However, in the 234432 arrangement the hydrophobic plane would be directly exposed to water, which causes a significant penalty of water entropy and, which is unlikely to be stable in a hydrated cell wall. As for the 1233321 arrangements (c and d), they expose the hydrophilic face instead of the hydrophobic surface, which is more compatible with hydrated conditions (Figure S6). The choice of the molecular arrangement inside the 18-chain microfibril depends on the hypothesis behind the interpretation of “interior” and “surface” peaks, which yields the range of interior cellulose chain numbers.

How the cellulose elementary fibrils interact with the surrounding matrix, particularly the hemicellulose is important to understand the cell wall structure, because they coexist at the genesis. Without hemicellulose, cellulose microfibrils would stick to each other forming larger microfibrils, as seen in cell walls with high cellulose content such as cotton fibers. Previous ssNMR reported that 2-fold xylan intimately binds with cellulose microfibril in *Arabidopsis* (Eudicot)^38^ and Pines (Conifer).^73^ The 2-fold xylan shows subnanometer contact to the cellulose surface, placing both the 2-fold xylan and surface cellulose in proximity to water molecules. Thereby, they exhibit similar dynamic behavior for both surface cellulose and 2-fold xylan, as evidenced by ssNMR.

In wheat, lignin domains can be considered as two distinct parts due to their even distribution between rigid and mobile regions. One population exhibits a dynamic feature similar to those reported previously,^74^ suggesting that lignin may assemble into water-repelling nanodomains. The remaining lignin interacts with polysaccharides in the secondary cell wall.^37,41,42^ In previous studies, cellulose microfibrils are primarily coated with hemicellulose, followed by the successive deposition of other hemicellulose and lignin.^75^ Our NMR data on wheat straw further reveal the existence of cellulose and lignin/xylan cross peaks within subnanometer distances (Figure 6b,c; regions 2 and 3). Collectively, the model suggests that the small interfibril spaces can bring the matrix polymers into spatial proximity with cellulose even in the absence of attractive interactions. These are prompting a reassessment of the specific interaction between the matrix polymers and the cellulose microfibrils.

## Conclusions

A new approach to construct an explicit model of an extended and highly crowded secondary cell wall based on the integrated use of X-ray scattering and ssNMR data sets was developed. A random lateral arrangement of 18-chain cellulose microfibrils with a nonpenetrating surface layer explains the X-ray small-angle scattering profile. The arabinoxylan in wheat behaves differently from the glucuronoxylan in *Arabidopsis*, and does not tightly bind to cellulose despite forming 2-fold helical conformation. This is also the first indication that the 2-fold conformation does not necessarily require tight binding to the flat surface of a cellulose microfibril. This approach can be further used to existing data on other plant cell walls, and future studies of such complex biosystems.

## Methods

### Preparation of ^13^C-Labeled Wheat Straw

Uniformly ^13^C-enriched wheat straw (U-60416, 95 atom % ^13^C) was obtained from IsoLife bv (Wageningen, The Netherlands), cultivated from seed to harvest under controlled conditions. In brief, wheat plants (*T. aestivum*) were uniformly labeled with ^13^C to 95 atom % at 16 weeks postsowing. Plants were cultivated hydroponically in custom-designed, airtight labeling chambers of closed atmosphere and high-irradiance conditions. The photosynthetic photon flux density (PPFD) was 800 μmol m^–2^ s^–1^ at plant canopy height, with a 16 h light and 8 h darkness cycle. Ambient temperatures within the chambers were maintained at 24 °C during the day and 16 °C at night, with relative humidity levels at 75% during the day and 80% overnight. An average concentration of 400 ppm ^13^CO_2_ was kept during the illumination period was maintained by injection from pressurized gas cylinders. Nutrients and water were supplied through aerated Hoagland-type solutions supplemented with essential micronutrients and iron, with nitrogen concentrations kept within the range of 25–200 mg L^–1^, pH values between 5.0 and 6.5, and electrical conductivity between 0.4 and 0.7 mS cm^–1^. Immediately following harvest, plant tissues were carefully separated; leaves and stems were sectioned into smaller fragments and rapidly stored at –30 °C prior to freeze-drying (lyophilization). The lyophilized samples were preserved under dark and dry conditions at 18 °C.

### Solid-State NMR Experiments and Analysis

The wheat straw was hydrated to approximately 40 wt %, after which 30 mg of the material was cut into slices and loaded into a 3.2 mm Bruker MAS rotor for ssNMR experiments.^76^ Solid-state ^13^C MAS NMR experiments on wheat straw samples were performed using a 3.2 mm Bruker E-free probe on an 800 MHz Bruker Avance Neo spectrometer at 295 K with a magic angle spinning (MAS) frequency of 16–19 kHz. ^13^C chemical shifts were externally calibrated using the CH_2_ signal of adamantane, set at 38.48 ppm on the tetramethylsilane (TMS) scale. Typical radio frequency field strengths employed during the experiments ranged from 62.5 to 83.3 kHz for ^1^H and 50 to 71.4 kHz for ^13^C.

Resonance assignment of polysaccharide and lignin signals were performed mainly via 2D refocused J-INADEQUATE (Incredible Natural Abundance Double Quantum Transfer Experiment) spectroscopy.^77,78^ This technique utilizes correlations between single quantum (SQ) spin pairs in the direct dimension and their chemical shift sum in the double quantum (DQ) indirect dimension to achieve enhanced resolution of overlapping signal pairs in two-dimensional space. J-based coherence transfer selectively highlights spin pairs of two covalently bonded carbons.^79,80^ Resonance assignments were conducted with the assistance of the established Complex Carbohydrate Magnetic Resonance Database (CCMRD) and pertinent literature.^81^

Rigid cell wall components were detected via Cross-Polarization (CP)^82^ based experiments. Typically, a 1 ms ^1^H–^13^C CP contact time was used to excite all rigid components. ^13^C direct polarization (DP) with a short recycle delay of 2 s was utilized to preferentially detect relatively mobile molecules that have rapid ^13^C-T_1_ relaxation.

Intramolecular interactions (≤6 Å) were detected via 2D ^13^C–^13^C Dipolar-Assisted Rotational Resonance (DARR)^83^ experiment with a short mixing time of 80 ms. Intermolecular contacts (up to 1 nm) were identified using 2D ^13^C–^13^C Proton-Driven Spin diffusion (PDSD)^84^ with a mixing time of 1 s. To enhance the detection of lignin signals in the presence of proton-rich polysaccharides, a modified version of DARR and PDSD experiments (gated-DARR and gated-PDSD)^85,86^ was employed with an addition of a gating period, which reintroduces^13^ C–^1^H dipolar coupling and selectively dephases signals of protonated carbon sites in the indirect dimension. This dipolar dephasing period was asymmetrically placed relative to the π pulse in the Hahn echo sequence to maximize the dephasing, incorporating two uncoupled delays of 32 and 16 μs. Cross-peaks of wheat straw were identified and integrated using a ±1 ppm window around the corresponding chemical shift and categorized into strong (>4.0%), medium (2.0–4.0%), and weak (<2.0%), based on the relative intensities relative to the total integration of one-dimensional slices over 10–180 ppm. To visualize the molecular interactions, we constructed a chord graph. Interactions were classified into three categories—strong, medium, and weak—and assigned values of 3, 2, and 1, respectively, to represent their intensity in the graph.

The hydration profile of biomolecules was determined using the water-edited experiment.^65,66^ A ^1^H-T_2_ relaxation filter of 1 ms × 2 was used to suppress the polysaccharide signals to <5% of the original intensity, while retaining the majority of water magnetization (>85%). The polarization of ^1^H from water was further transferred to nearby molecules using a 2.25 ms ^1^H mixing period, followed by a 1 ms ^1^H–^13^C CP transfer. Both the water-edited spectrum and the control spectrum were acquired with an 80 ms ^13^C–^13^C DARR mixing period. One-dimensional water-to-polysaccharide/lignin buildup curves were generated using a ^1^H-T_2_ filter of 1 ms × 2, along with ^1^H mixing times ranging from 0 to 100 ms^65^. These *S*/*S*_0_ intensity ratios, which compare the intensities between the water-edited spectrum (S) and the control spectrum (S_0_), indicate the degree of water retention around various carbon sites in the cell wall.

^13^C-T_1_ relaxation measurements were conducted using two approaches. The CP-based Torchia T_1_ technique and the standard DP-based inversion recovery were used.^87^ The Torchia-CP preferentially detected rigid molecules in its z-filtered versions, while the inversion recovery experiment with a recycle delay of 30 s was used to quantitatively probe all molecules. Relaxation time constants were determined by fitting using single exponential functions.

Dipolar order parameter (S_CH_) was calculated from C–H dipolar couplings measured using the dipolar-chemical-shift (DIPSHIFT) experiment at 7.5 kHz MAS was used. Spin-diffusion suppression was employed in the ^1^H-T_1_ρ measurements to mitigate proton magnetization equilibration across nanoscale domains, which would otherwise obscure component- and site-specific relaxation behavior. ^1^H-T_1ρ_ relaxation was detected using 62.5 kHz for the Lee–Goldburg spin-lock sequence,^88^ with the scaling factor confirmed to be 0.577. CP-DIPSHIFT experiments were performed with a recycle delay of 2 s, while quantitative DP-DIPSHIFT experiments were conducted with a recycle delay of 20 s.

### Solution-State NMR Experiments

Approximately 50 mg of uniformly 13C-labeled wheat straw was cut into small pieces and ground in a clean porcelain mortar with a pestle for about 1 h to obtain a fine, homogeneous powder. The material adhering to the mortar wall was periodically scraped down with a spatula and reincorporated to minimize loss. The resulting powder was collected by scraping and transferred into a preweighed vial. Subsequently, approximately 20 mg of the sample was directly dissolved in 0.5 mL of dimethyl sulfoxide-*d*_6_ (DMSO-*d*_6_), followed by ultrasonication for 60 min at 25 °C to ensure a homogeneous mixture before measurement. After sonication, the mixture was immediately transferred into a 5 mm NMR tube for measurement. The central solvent peaks (δH/δC 2.50/39.52) were used as internal references. The dissolved sample was directly transferred into a 5 mm NMR tube for solution NMR analysis. The 2D ^1^H–^13^C HSQC was conducted at 289.2 K on a Bruker AVIII 400 MHz spectrometer equipped with a 5 mm BBO probe. The ^1^H and ^13^C dimensions were each acquired with 1024 points, corresponding to acquisition times of 13200 and 5000 Hz in the F2 and F1 dimensions, respectively, with a recycle delay of 1 s. A total of 64 transients were collected, and 256 increments were recorded in the ^13^C dimension. The methods for calculating semiquantitative values for lignin units are based on procedures outlined in the literature.^89^ The S_2,6_, G_2_, and H_2,6_ signals were used to represent S, G, and H units, respectively, and the integrals of the S and H signals were divided by two.

### X-Ray Scattering

X-ray scattering was measured using Xeuss 3.0 (Xenocs, France) equipped with a Pilatus 3R 300 K detector positioned at 0.085 m for WAXS and 0.3 m for SAXS. The beam size was 100 μm. A thin piece of wheat straw sample with controlled water content (5 mm × 5 mm × 1 mm) was sandwiched between Kapton adhesive tapes and attached to the sample holder, and exposed for 300 s. Two measurements with the detector vertically translated were performed for each sample to fill the gap zone. The scattering from the air in the sample chamber, windows, and Kapton films was measured without the sample, and subtracted from the data after normalization with the transmitted intensity of the direct beam (the apparatus has no beamstop). Scherrer crystallite size is defined by *L* = 2π*K*λ/*B*, where *K* is the form factor and is set as 0.90,^90^ λ is the X-ray wavelength, 1.542 Å, B is the full width at half-maximum (fwhm) of the diffraction peak.

### Scattering Model

The measured small-angle intensity *I* (*q*) was modeled as a background-offset product of structure S (*q*) and a form factor *P* (*q*)­

$$I\left(q\right)=AS\left(q;\phi ,qR_{e}\right)P\left(q;R\right)+B$$

where *q* is the amplitude of the scattering vector (Å^–1^), *R* is the radius, *R*_e_ is the effective radius with a sheath layer *R*_e_ *–R* (Å), ϕ is the effective volume fraction including the sheath volume as part of the cylinder, *A* is an overall scale factor, and *B* is a flat background.

The structure factor S­(*q*; ϕ, *R*) was evaluated from a precomputed B-spline surface stored in “bsp.pickle” given by Nishiyama 2025 (ref 91), available at https://github.com/yoshi-CERMAV/hard_disk_rdf. The spline encodes S on a grid of (ϕ, q) generated previously on the basis of a unit radius of the cylinder. We evaluate it using Scipy’s bisplev

S(q;ϕ,R)=bisplev(qR,ϕ,bsp)

The cellulose microfibrils are approximated as infinitely long cylinders, whose form factor amplitude is proportional to J_1_(*qR*)/(*qR*), where J_1_ is the first-order Bessel function of the first kind, and

$$P\left(q;R\right)={\left(J_{1}\left(qR\right)/\left(qR\right)\right)}^{2}$$

## Supplementary Material



## Notes

Additional methods and analyses (Notes S1–S4), supplementary NMR/X-ray scattering and compositional data (Figures S1–S12; Tables S1–S9), and references (PDF)
