# Supplementary material for: Native Architecture of Wheat Straw Cell Walls: A Unified Model from X‑ray Scattering and Solid-State NMR
Source: J Am Chem Soc. 2026 Mar 19;148(20):20448–63. doi: 10.1021/jacs.5c23116 (PMC13220300; doi:10.1021/jacs.5c23116)
Supplement: Supplementary file 1 [file ja5c23116_si_001.pdf]

Supporting information for

Native Architecture of Wheat Straw Cell Walls: A Unified Model from X-ray Scattering and Solid-State NMR

Yucheng Hu[1], Pan Chen[2], Peng Xiao[3], Shixu Yu[1], Lingfeng Zhou[2], Zhe Ling[4], Yutong Zhu[1], Guohua Miao[1], Yuan He[1], Haichao Li[1], Sheng Chen[1], Tingting You[1], Feng Xu[1, *], Tuo Wang[3, *], Yoshiharu Nishiyama[5, *]

[1] State Key Laboratory of Efficient Production of Forest Resources, Beijing Key Laboratory of Lignocellulosic Chemistry, Beijing Forestry University, Beijing 100083, China
[2] School of Materials Science and Engineering, Beijing Institute of Technology, 100081 Beijing, China
[3] Department of Chemistry, Michigan State University, East Lansing, MI 48824, USA
[4] Jiangsu Co-Innovation Center of Efficient Processing and Utilization of Forest Resources, College of Chemical Engineering, Nanjing Forestry University, Nanjing 210037, China
[5] Univ. Grenoble Alpes, CNRS, CERMAV, 38000 Grenoble, France

*E-mail: xfx315@bjfu.edu.cn (F. Xu); wangtuo1@msu.edu (T. Wang); yoshiharu.nishiyama@cermav.cnrs.fr (Y. Nishiyama)

This PDF file includes:

Notes S1 to S4
Figure S1 to S12
Table S1 to S9
References (1 to 6)

Table of Contents

Note S1:..3
Note S2:..4
Note S3:..5
Note S4:..6
Figure 7
Figure S1. Quantitative analysis of carbohydrates. 7
Figure S2. ^{13}C DP-INADEQUATE spectrum of wheat straw. 8
Figure S3. HSQC analysis of lignin and polysaccharides. 9
Figure S4. Integration regions used for composition analysis in the CP-INADEQUATE spectrum. 10
Figure S5. Composition analysis from intramolecular interactions detected via DARR. .11
Figure S6. Range of interior cellulose chain numbers as a function of cross-sectional arrangements. 12
Figure S8. ^{13}C-^{1}H dipolar order parameters of biopolymers for CP and 40 s DP. 15
Figure S9. NMR relaxations of cell wall components. 16
Figure S10. Water-to-biomolecule polarization transfer buildup curves. 17
Figure S11. Water accessibility probed by 2D ssNMR spectra. 18
Figure S12. The interaction of polymers in the cell wall. 19
Table 21
Table S1. ^{13}C chemical shifts of rigid molecules. 21
Table S2. The molar composition of cellulose and xylan. 22
Table S3. ^{13}C chemical shifts of mobile molecules. 23
Table S4. The crystalline structure of fibril (n=3). 24
Table S5. The dipolar order parameter S_{CH}. 25
Table S6. ^{1}H-$T_{1\rho}$ relaxation times of lignin and polysaccharides. 26
Table S7. ^{13}C-T_1 relaxation times of lignin and polysaccharides. 27
Table S8. Water-edited intensities of biopolymers. 28
Table S9. Intermolecular interactions of polymers. 29
References 30

Note S1:

Calculation of Wet-Basis Mass and Volume Fractions

The sample consists of 64.72 wt% carbohydrates, 18.5 wt% acid-insoluble (AI) lignin, and 1.5 wt% acid-soluble (AS) lignin The monosaccharide molar percentages within the carbohydrate fraction are given as: Arabinose (3.84%), Galactose (0.97%), Glucose (53.24%), Xylose (37.37%), and Uronic acid (4.22%) in ref 1. The molar masses and densities employed in the calculation are :

Molar masses ($g{\cdot}mol^{-1}$): Arabinose 132.116; Galactose 162.141; Glucose 162.141; Xylose 132.116; Uronic acid 176.124; Water 18.0.

Densities ($g{\cdot}cm^{-3}$) of 1.60 (cellulose), 1.52 (hemicellulose), 1.30 (lignin), and 1.00 (water). For cellulose, 1.60 $g{\cdot}cm^{-3}$ is the crystallographic density of ideal cellulose I (1.63 $g{\cdot}cm^{-3}$);[2] since our measured 200 d-spacing is ~5% larger, the effective cellulose density in the sample is reduced by ~5% when converting mass to volume fractions. The hemicellulose fraction is dominated by arabinoxylan, so we take 1.52 $g{\cdot}cm^{-3}$ as the hemicellulose density, consistent with reported crystalline D-xylose densities[2],

Conversion of mol% to mass fractions in carbohydrates

For each sugar component i, the mass fraction is calculated as: $wi = (n_i \times M_j) / (\sum_j (n_j \times M_j))$, where is the mol% of sugar *i*, and is its molar mass.

The calculated carbohydrate mass fractions are: Arabinose 0.0339; Galactose 0.0105; Glucose 0.5764; Xylose 0.3296; Uronic acid 0.0496.

Wet-basis mass fractions

The solid mass fraction is converted into wet-basis values with moisture content of 30% by dividing with 1.3

Resulting mass fractions: Cellulose 48.5%; Hemicellulose 35.64% (Arabinose 2.04%; Galactos 0.63%; Uronic acid 2.98%); Lignin 26%; Water 23%.

Wet-basis volumes

Volumes are obtained from the wet-basis masses divided by corresponding densities: $V_i = m_i / p_i$

The unnormalized volumes are: Cellulose 30.3 cm^3; Hemicellulose 24.6 cm^3; Lignin: 20 cm^3; Water: 30 cm^3.

Normalized wet-basis volume fractions

The volume fractions are normalized over the four components: $\phi_i = 100 \times (V_i / \sum_j V_j)$

Resulting values: $\phi_{Cellulose} = 29\%$; $\phi_{Hemicellulose} = 23\%$; $\phi_{Lignin} = 19\%$; $\phi_{Water} = 29\%$; $\phi_{Sum} = 100.00\%$.

Note S2:

Estimation of the numbers of two-fold and three-fold xylan chains per 18-chain cellulose microfibril.

In the rigid carbohydrate fraction probed by DP ssNMR (40 s recycle delay), the total contribution of cellulose plus xylan is 12% based on the integration of C1, two-fold while the beta 1, 4 linked pyranose represents 10% based on the C4 integration >80 ppm. According to sugar analysis, cellulose and xylan account for 53.24% and 37.37% of the total carbohydrate molar fraction, respectively, corresponding to 7% cellulose and 5% xylan within this 12% carbohydrate fraction.

The whole 7% of cellulose takes the two-fold helical conformation, so the remaining 3% would be xylan forming the two-fold helix,. Since 5% was xylan, (5 – 3)% would be the three fold xylan. So the three-fold:two-fold ratio within xylan is 2:3.

Note S3:

To estimate the equivalent cylindrical radius of an 18-chain cellulose microfibril, take a slice one cellobiose long (two glucose residues; $L \approx 10.3$ Å). Its mass is $(18 \times 2 \times 162 \text{ g mol}^{-1})/N_A$; dividing by the crystalline cellulose density ($\rho \approx 1.6 \text{ g cm}^{-1}$) gives a volume of ~ 6050 Å^3. The corresponding radius $r = (A/pi)^{1/2} \approx 13.7$ Å.

Note S4:

We simulate a 2D hard-disk fluid under periodic boundary conditions at a target area fraction ϕ using Metropolis Monte Carlo with strict overlap rejection. For N disks of radius R, the square box length L is set by

$L = sqrt((N \times pi \times R^2) / \phi)$

At each step, a random disk is displaced; PBC are handled via the minimum-image convention.

To quantify void space, we place a regular mesh with spacing 0.1R over the box. For each mesh point x, we compute the nearest-center distance $d_{min}(x)$ (with PBC) and the gap to the disk surface.

$g(x) = d_{min}(x) - R$

We report the normalized histogram of g and save the final configuration, metadata, and histogram data for reproducibility.

Figure

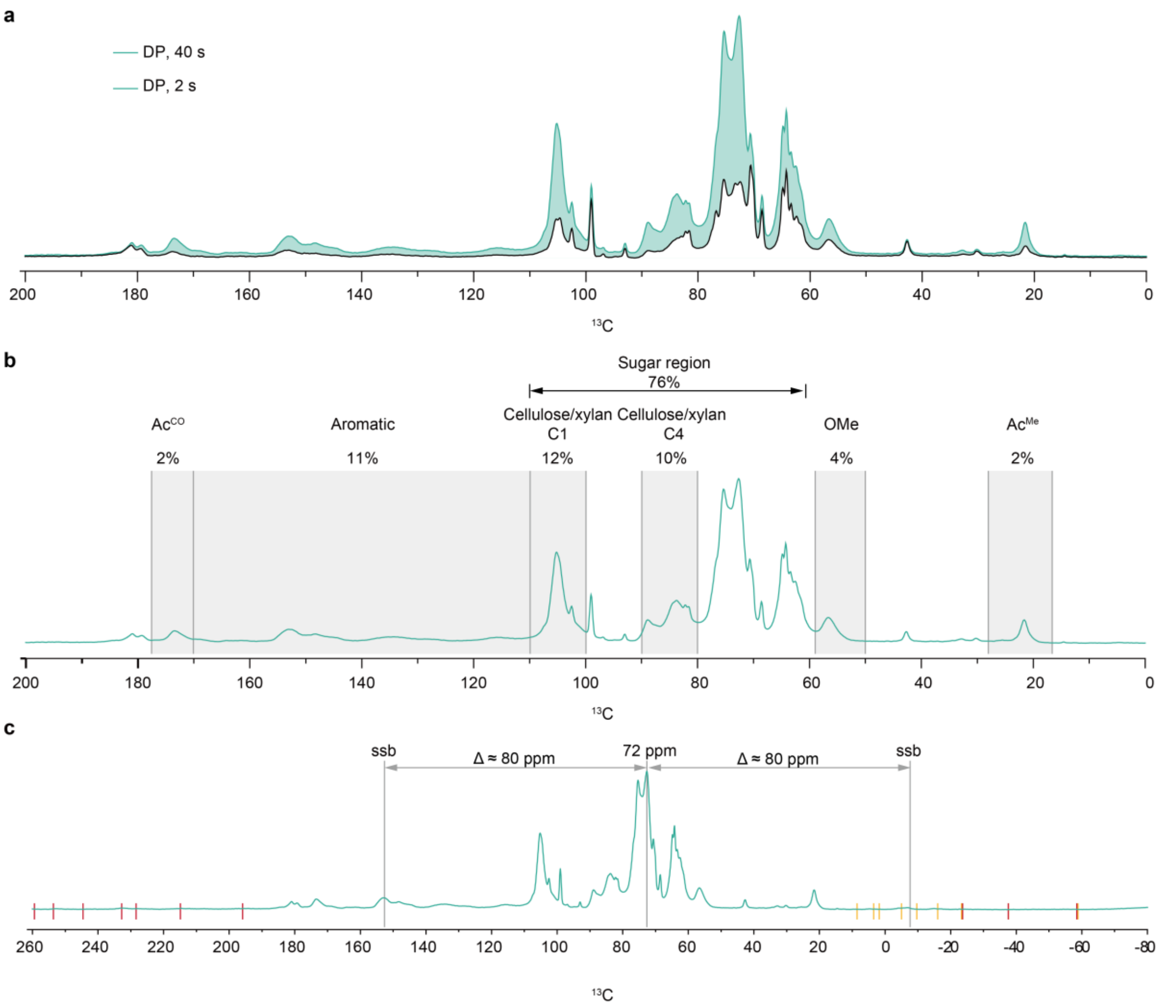

Figure S1. Quantitative analysis of carbohydrates. a, Difference of integration obtained by subtracting the DP-2s spectrum from the DP-40s spectrum. **b**, integration regions defined for the DP-40s spectrum. Error bars estimated from the RMS noise (σ) measured in a signal-free region (45-52 ppm) are only 0.01-0.02% (absolute population), but uncertainties arising from the choice of integration range and peak overlap may introduce error margins of a few percent of the integrated values. c, The spinning side band (SSB) was labeled. Although SSBs can overlap with the lignin (orange) and polysaccharide (red) integration windows, integration of the major SSB contributions indicates that they account for ~2% of the lignin-window intensity and ~0.1% of the polysaccharide-window intensity; therefore, their effects on the semiquantitative populations are negligible and no sideband correction was applied.

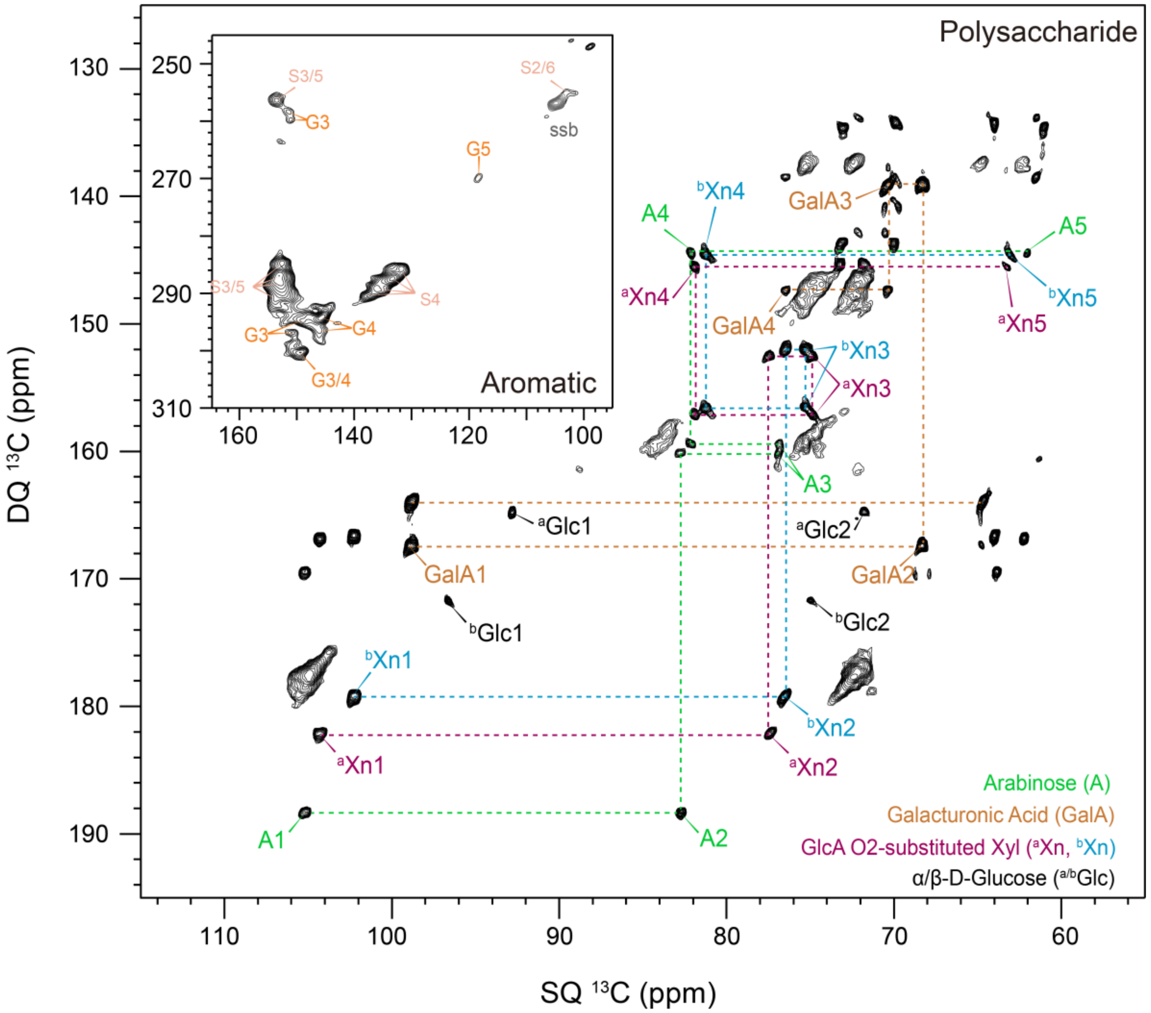

Figure S2. ^{13}C DP-INADEQUATE spectrum of wheat straw. The relatively mobile molecules were selected by DP ^{13}C excitation with a short recycle delay of 2 s. The polysaccharide region is shown in the main panel, with the identified components listed. Full carbon connectivity of these components is linked by colour-coded dashed lines. The aromatic region is shown in the inset. Only S and G lignin were detected in the mobile fraction, and no mobile ferulate was detected.

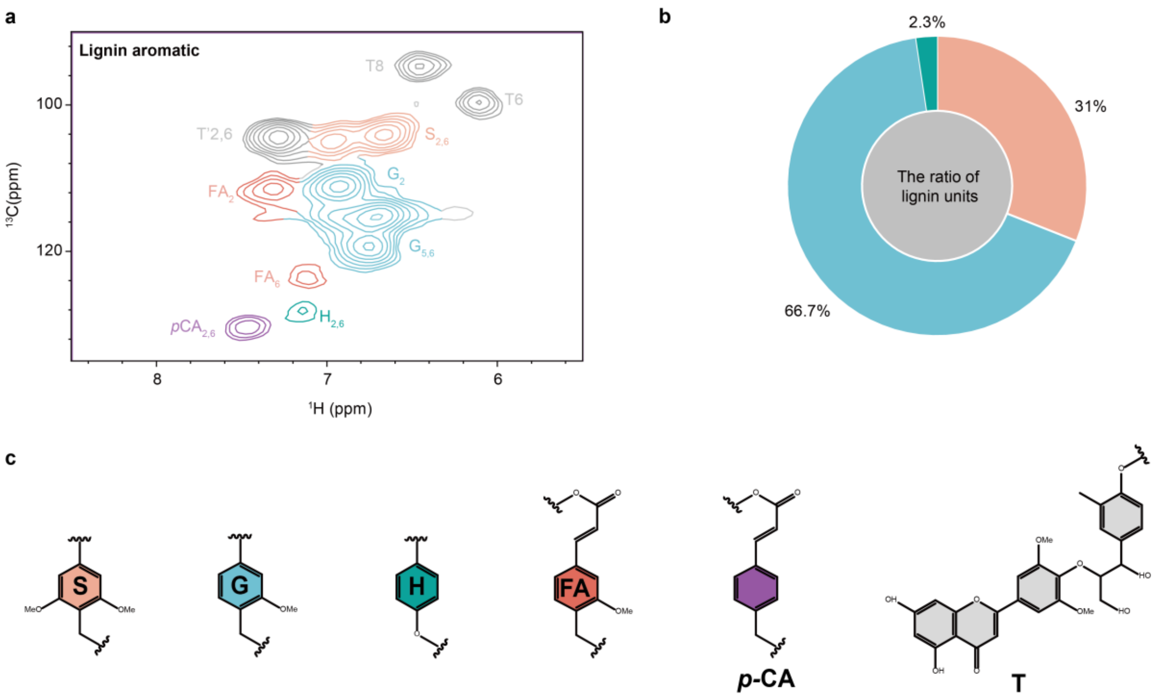

Figure S3. HSQC analysis of lignin and polysaccharides. **a**, lignin aromatic region; **b**, the ratio of lignin determined from the HSQC spectrum. **c**, Illustrates the structure of lignin units. Main structure [3]: (S) syringyl units; (G) guaiacyl units; (H) *p*-hydroxyphenyl units; (*p*-CA) p-coumarates; (FA) ferulates; (T) a likely incorporation of tricin into the lignin polymer through a G-type β-O-4 linkage.

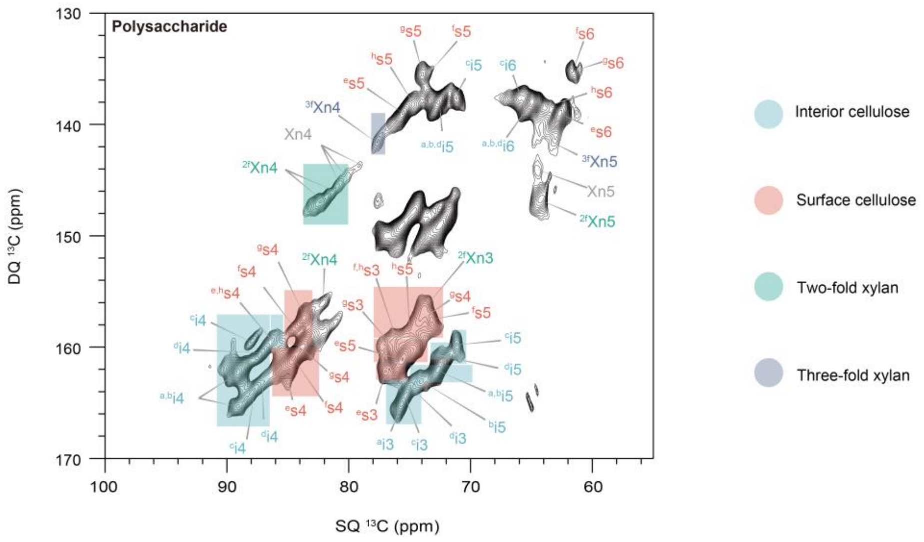

Figure S4. Integration regions used for composition analysis in the CP-INADEQUATE spectrum. The cellulose region (C3–C4–C5) was integrated to estimate the interior (light blue) vs surface (light pink) cellulose fractions. The xylan C4 region was integrated to quantify the 2-fold and 3-fold xylan populations.

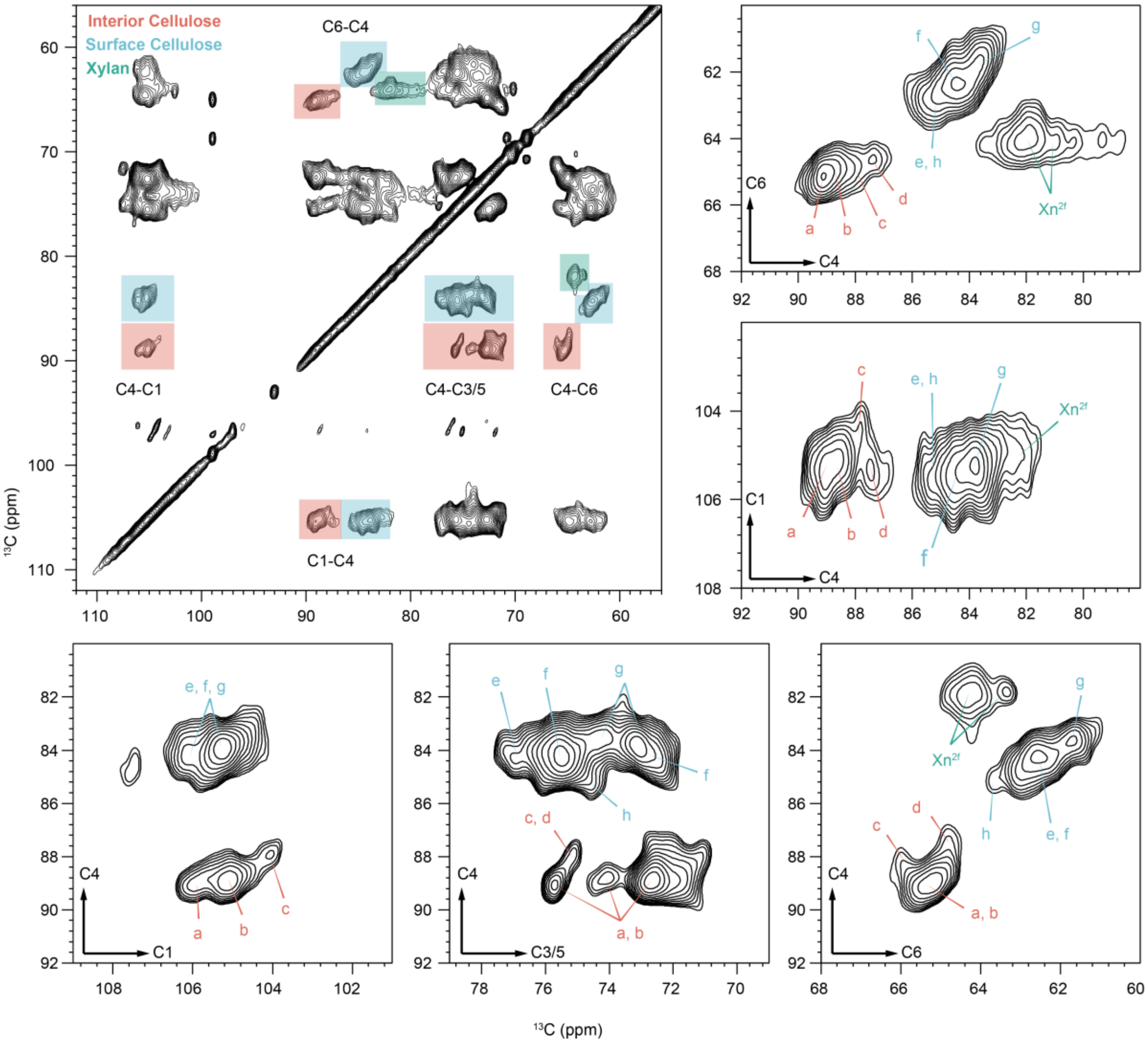

Figure S5. Composition analysis from intramolecular interactions detected via DARR. The cross-peaks in the DARR experiment with 80 ms mixing preferentially exhibit carbon correlations within a few bonds and certain unique cross-peak areas, e.g., C1-C4, C4-C3/5, and C4-C6, can be unambiguously distinguished for surface cellulose (blue), interior cellulose (red), and 2f xylan (green). The highlighted peak areas in colour were used to calculate their molecular populations for composition analysis. The zoomed-in panels show additional details for different forms (a-g) of each type of polysaccharide unit, most of which are well-resolved in the DARR spectrum.

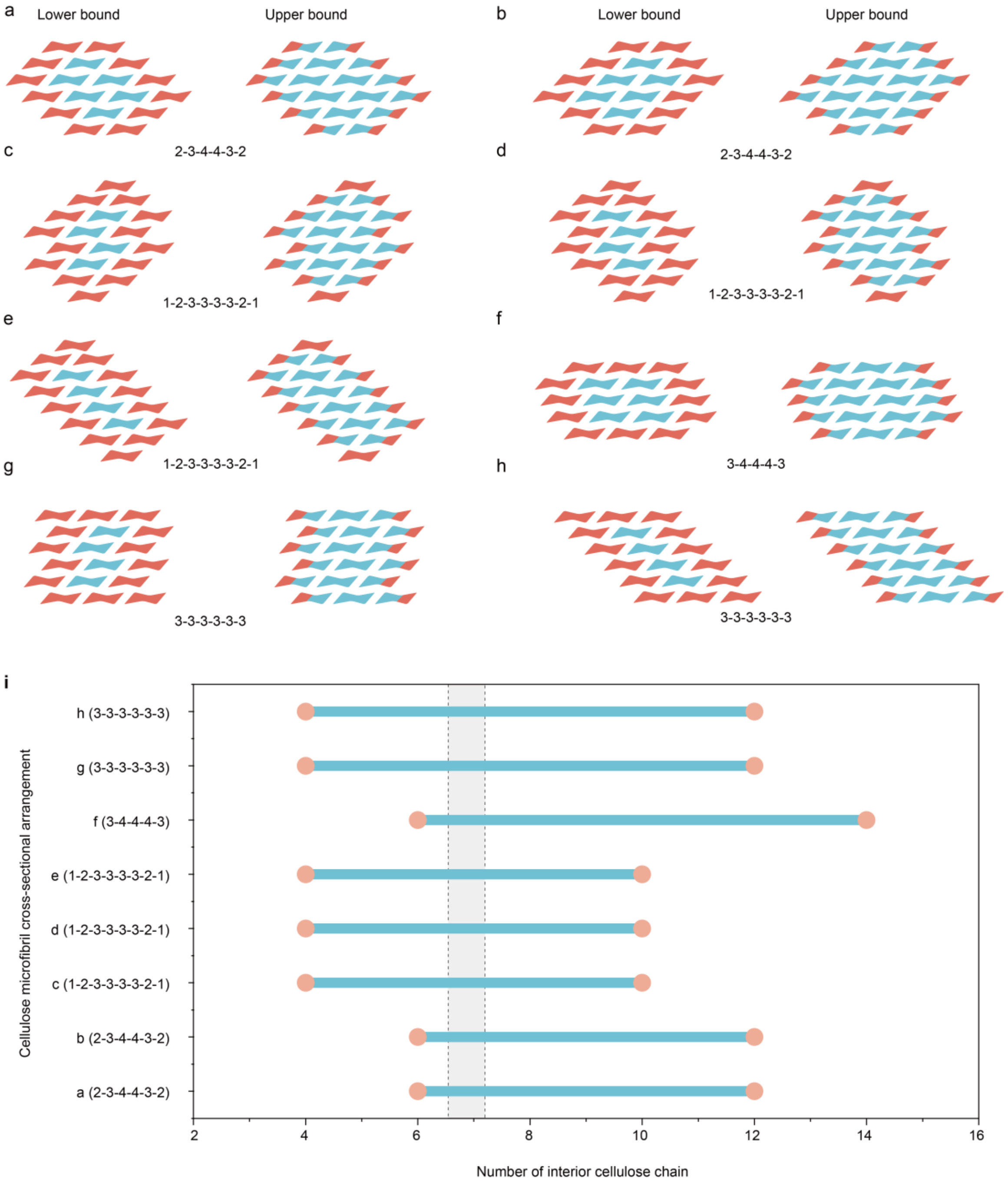

Figure S6. Range of interior cellulose chain numbers as a function of cross-sectional arrangements. Each arrangement shows the upper and lower bounds of the number of interior chains. The upper bound corresponds to the originally defined interior chains, whose C6 hydroxymethyl groups adopt the *tg* conformation. The lower bound additionally assumes that some outer chains, whose C6 groups point toward the fibril interior, contribute to the interior-like population. For example, in arrangement **a** (234432), the right blue-marked chain denotes the lower bound of the interior chains count, whereas the left ones

denote the upper bound. Type a-h shows different cross-sectional arrangements based on 18-chains, which have various hydrophilic and hydrophobic faces. The bottom panel lollipop plot standard for the range of interior cellulose chain numbers as a function of cross-sectional arrangements. The two points in the lollipop plot represent the upper and lower bounds of the number of interior chains corresponding to each arrangement. The grey band indicates the range of interior chain numbers estimated based on CP-INADEQUATE and DARR experiments (**Table S2**), highlighting candidate microfibril cross-sectional arrangements that satisfy the experimentally measured cellulose chain number range.

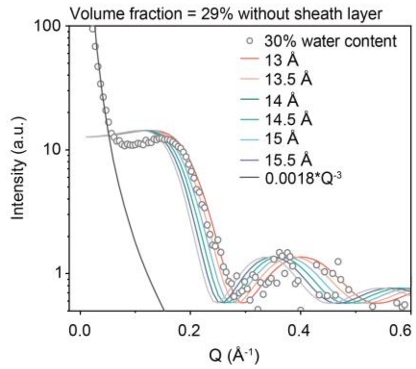

Figure S7. Simulated scattering profile of 29% randomly packed parallel cylinder with different diameters. Comparison of X-ray small-angle scattering data with calculated structure factors and the form factor assuming a random packing of infinitely long cylinders with various radius (13, 13.5, 14, 14.5, 15, 15.5 Å).

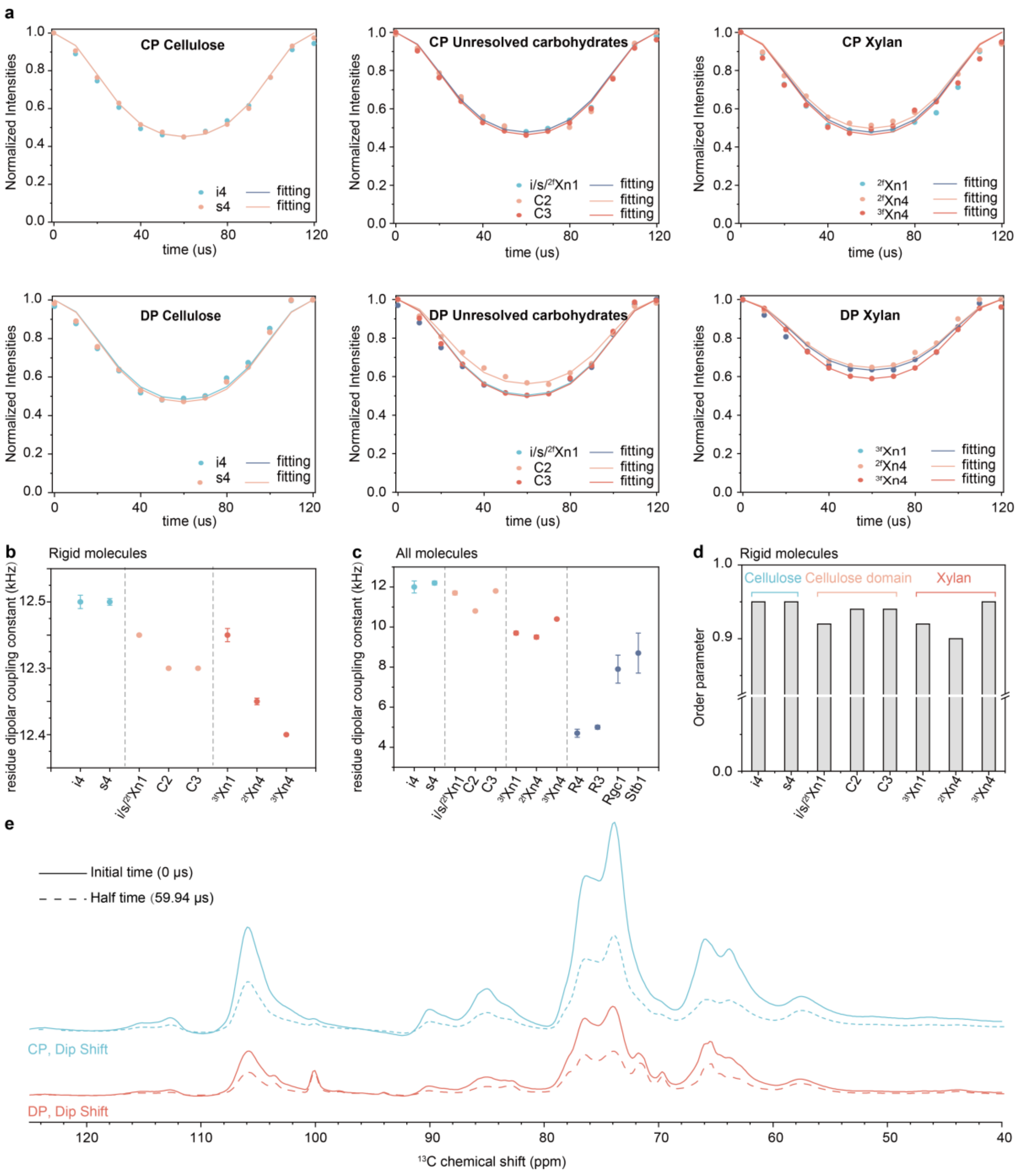

Figure S8. ^{13}C-^{1}H dipolar order parameters of biopolymers for CP and 40 s DP. a: CP-dipolar dephasing spectra (top panel) and 40 s DP-dipolar dephasing spectra (bottom panel) of carbohydrates are shown. The residual/apparent dipolar coupling constants of rigid molecules **b** and **c**. **d:** The order parameter ^{1}H-^{13}C was analyzed using CP-DIPSHIFT MAS NMR analysis. **e:** Slices taken at the initial point (0 μs dipolar dephasing) and at half-time (59.94 μs dipolar dephasing) from the 2D DIPSHIFT experiment demonstrate the degree of spectral intensity reduction occurring at half a rotor period.

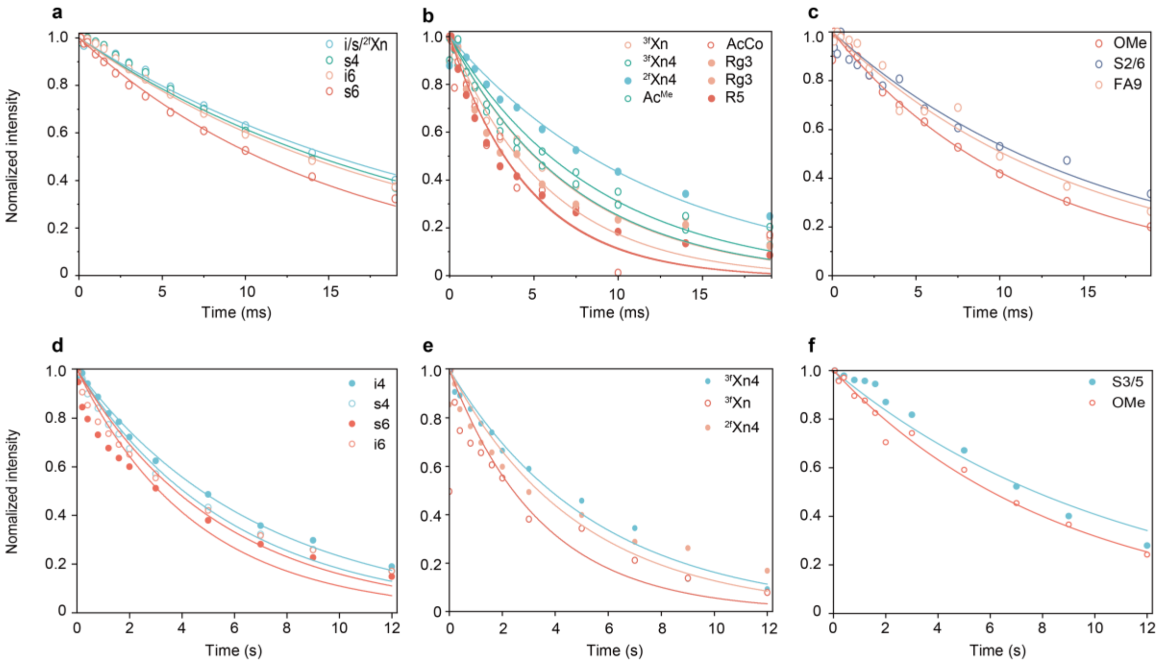

Figure S9. NMR relaxations of cell wall components. The ^{1}H-$T_{1\rho}$ relaxation (a-c) and ^{13}C-T_1 relaxation (d-e) with a single exponential fit. (**a**) cellulose (interior/surface glucan chains), (**b**) hemicellulose (two/three-fold xylan and other matrices), and (**c**) lignin. These relaxation data were fitted with a single exponential equation. The ^{13}C-T1 relaxation curves for (**d**) cellulose (interior/surface glucan chains), (**e**) hemicellulose (two/three-fold xylan), and (**f**) lignin were detected using Torchia CP.

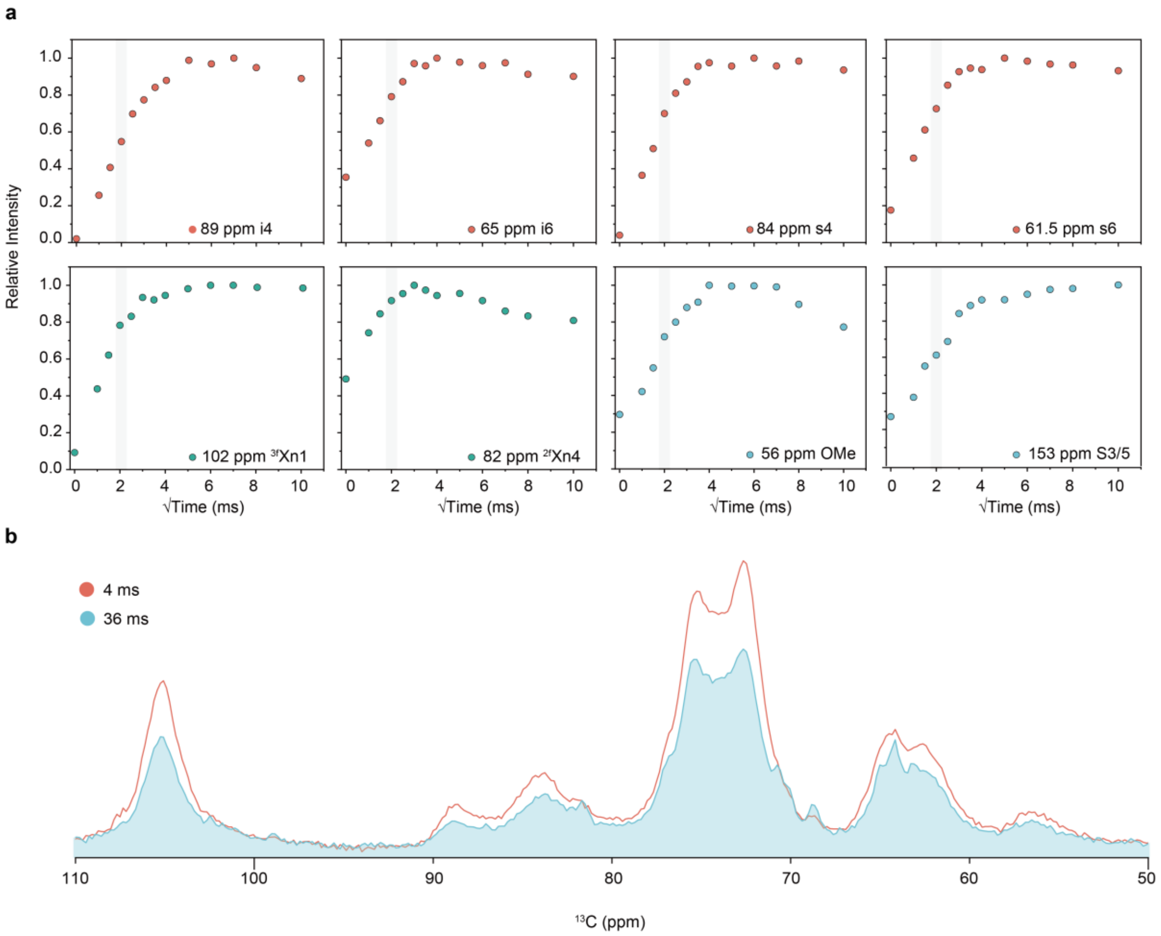

Figure S10. Water-to-biomolecule polarization transfer buildup curves. a, representative ^{1}H spin diffusion buildup curves for cellulose (i4, i6, s4, s6), xylan (^{3f}Xn1 and ^{2f}Xn4), and lignin (OMe and S3/5). The shaded vertical lines highlight the intensities of water-to-carbon transfer observed at 4-ms ^{1}H-^{1}H SD mixing. **b**, Overlay of 1D water-edited spectra with ^{1}H mixing times of 4-ms and 36-ms ^{1}H mixing time.

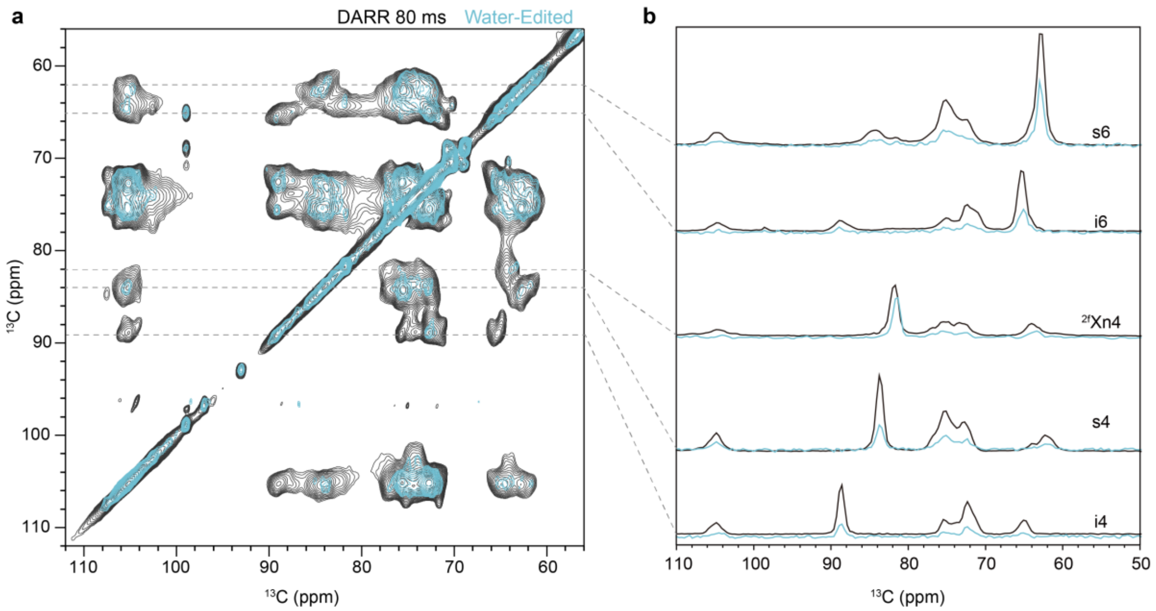

Figure S11. Water accessibility probed by 2D ssNMR spectra. a, 2D ^{13}C-^{13}C correlation spectra with the same 80 ms DARR mixing overlayed as: control spectrum (black) and water-edited spectrum (blue). **b**, Representative one-dimensional slices from water-edited (blue) and control (black) spectra.

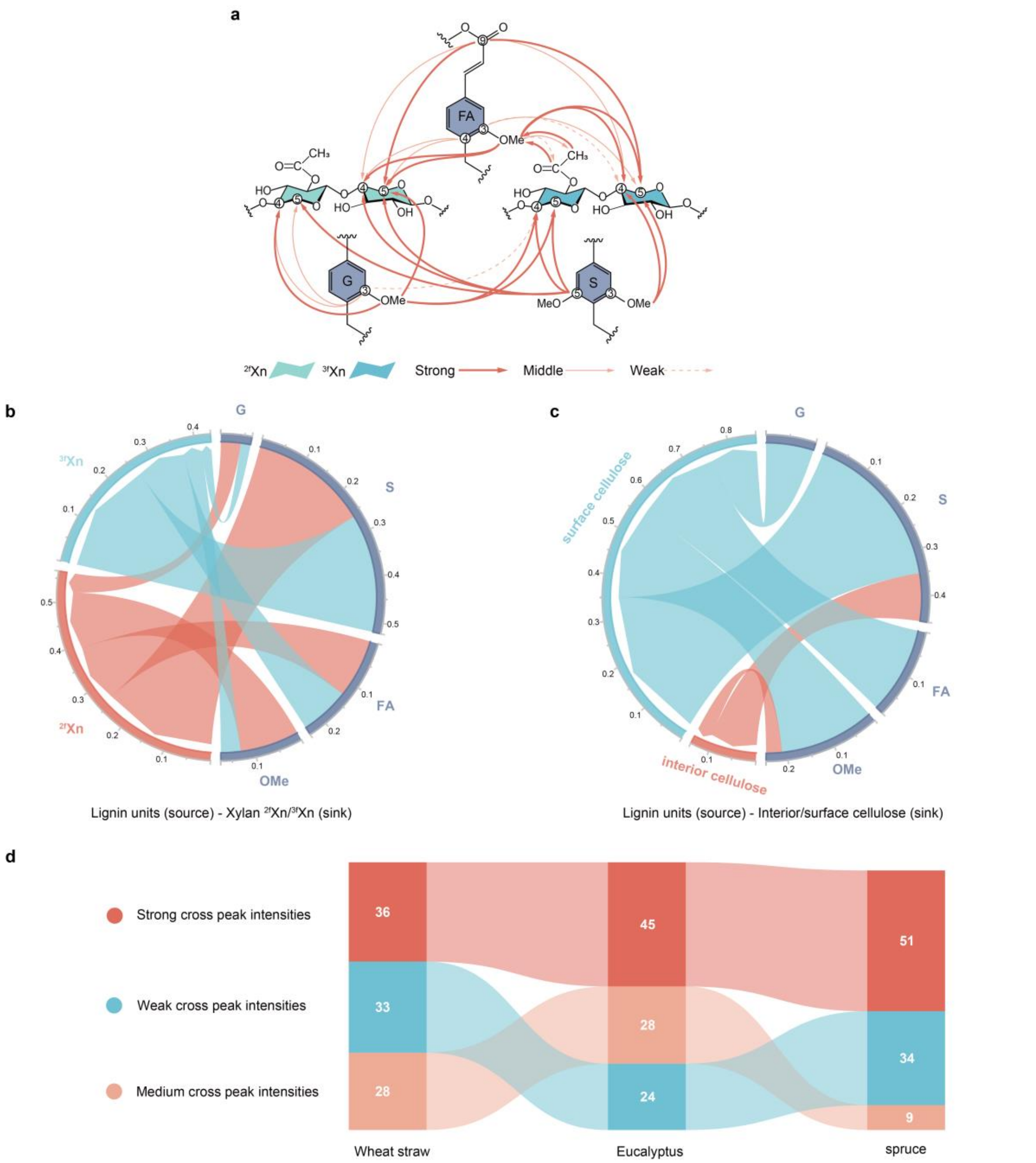

Figure S12. The interaction of polymers in the cell wall. NMR cross-peaks between lignin and xylan are expressed as red lines (Thick lines represent strong intensity, thin lines medium intensity, and dashed lines weak intensity). **b** and **c:** The cross-peaks among polysaccharide-lignin in the wheat straw; Each arc represents a source or sink, while the arrows connecting the circles illustrate the interactions between the two components. **d**, The intermolecular cross-peaks intensity from different lignocellulosic biomass [4]. The wheat straw shows relatively weak cross-peaks compared to other woody biomass (eucalyptus (*Eucalyptus grandis*) and spruce (*Picea abies*)).

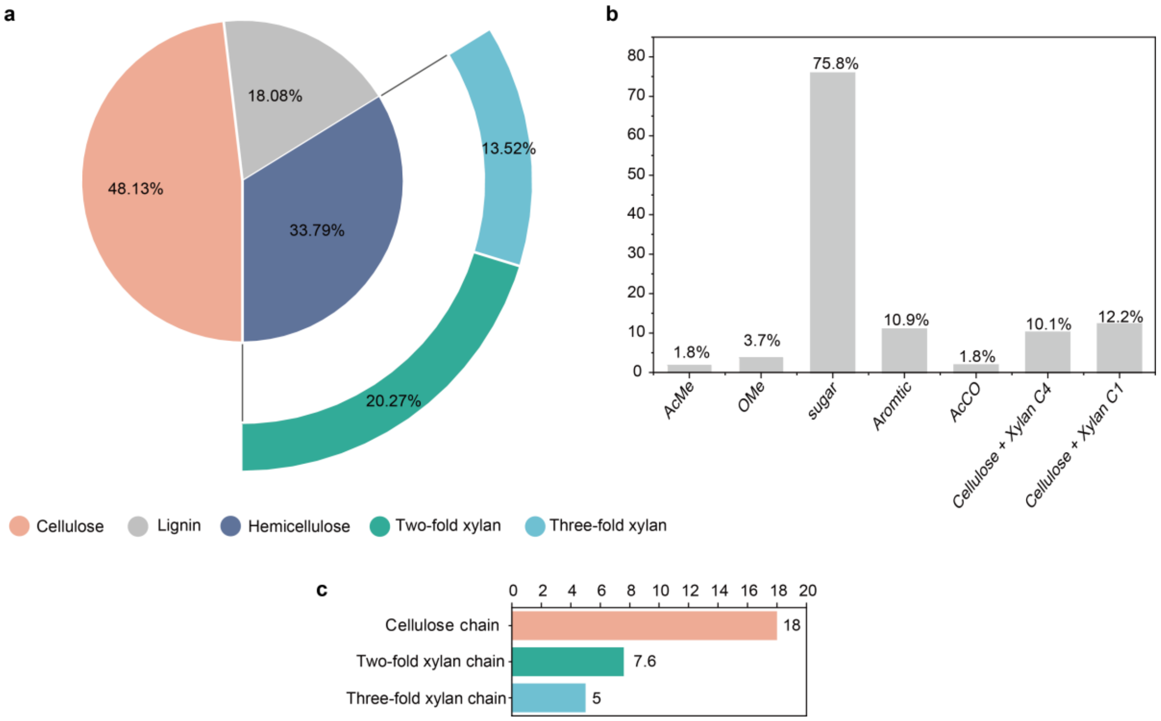

Figure S13 The composition of biopolymers in the cell wall. **a**, The chemical composition analysis from ref 1. **b**, The quantitative analysis from 1D DP 40 s ssNMR spectra. **c**, Chain number estimation as described in **Note S2**.

Table

Table S1. ^{13}C chemical shifts of rigid molecules. All components are identified from ^{13}C CP-based INADEQUATE spectra. Superscripts are used to denote different allomorphs. Not applicable (/). Unidentified (-).

CP-INADEQUATE	Type	C1	C2	C3	C4	C5	C6				Ref.
Cellulose	^{a}i	105.9	-	75.6	89.2	72.6	65.0	/	/	/	4,5
	^{b}i	105.1	-	74.1	88.8	72.6	65.0	/	/	/	
	^{c}i	104.0	-	75.1	87.9	71.1	65.8	/	/	/	
	^{d}i	105.4	-	75.6	87.4	73.2	64.5	/	/	/	
	^{e}s	105.6	-	76.9	85.2	75.6	62.6	/	/	/	
	^{f}s	105.6/106.1	-	76.0	84.4	73.2	62.5	/	/	/	
	^{g}s	105.2	-	75.7	83.7	73.8	61.7	/	/	/	
	^{h}s	105.6	-	75.5	85.4	74.7	63.6	/	/	/	
	**Type**	**C1**	**C2**	**C3**	**C4**	**C5**	**C6**	**AC^{CO}**	**AC^{Me}**		**Ref.**
Xylan	^{2f}Xn	104.2	75.4	73.8	82.1	64.2	/			/	6
	^{2f} Xn	104.2	75.4	74.6	81.1	64.6	/			/	
	Xn	102.6	-	74.6	81.1	64.6	/			/	
	Xn	-	-	76.5	81.1	64.6	/	173.7	21.4		
	Xn	-	-	76.8	80.1	64.3	/			/	
	Xn	-	-	74.6	79.2	64.5	/			/	
	^{3f} Xn	102.5	73.8	74.3	77.6	63.7	/			/	
	**Type**	**C1**	**C2**	**C3**	**C4**	**C5**	**C6**	**C7**	**C8**	**C9**	**Ref.**
Lignin	S_a	134.0	103.9	154.1	134.9	154.1	103.9	/	/	/	4
	S_b	-	106.7	154.3	137.5	154.3	106.7	/	/	/	
	G	-	109.5	151.9	147.9	115.6	-	/	/	/	
	FA	-	-	151.9	151.9	113.7	-	-	113.7	169.9	
	H	128.9	126.0	121.7	163.9	121.7	126.0	/	/	/	

Note: The assignment of chemicals can also refer to the Complex Carbohydrates Magnetic Resonance Database (CCMRD) [7].

Table S2. The molar composition of cellulose and xylan. Cellulose in these different chemical environments contains interior (i) and surface (s). Xylan in these different environments contains xylose chains (^{2f}Xn: two-fold xylan; ^{3f}Xn: three-fold xylan; Xn: mixed conformation).

**Cellulose**			
CP-INADEQUATE (C4)	Interior % 40	Surface % 60	interior cellulose chains 7.2
CP-INADEQUATE (C6)	Interior % 53	Surface % 47	interior cellulose chains 9.5
DARR	Interior % 37	Surface % 63	interior cellulose chains 6.6

**Xylan**		
CP-INADEQUATE (C4)	^{2f}Xn % 74	^{3f}Xn % 26

**Cellulose and xylan**				
CP-INADEQUATE	Interior % 36	Surface % 57	^{2f}Xn % 5	^{3f}Xn % 2

Note: The estimated cellulose ratios were cross-validated via two NMR methods with different polarization transfer pathways, the through-bond INADEQUATE method and the through-space DARR method (with a short mixing time of 80 ms). Both methods only emphasize the carbon correlations within the same molecule unit and each of their cross-peaks represents the population of a specific molecule unit. The discrepancy between the C4 (C4-C3/5 peaks) and C6 (C5-C6 peaks) derived values in INADEQUATE may due to potential overlap with other carbohydrate components in the cellulose C5-C6 peak regions.

The percertages were calculated were as follows:

$s\% = s/(s + i) \times 100\%$;

$i\% = i/(s + i) \times 100\%$;

$^{2f}Xn\% = {}^{2f}Xn/({}^{2f}Xn + {}^{3f}Xn) \times 100\%$;

$^{3f}Xn\% = {}^{3f}Xn/({}^{2f}Xn + {}^{3f}Xn) \times 100\%$.

Where s, i, ^{2f}Xn and ^{3f}Xn are the sum of all area integrations for each unit, illustrated in Figures S3 & 4. The source data was provided.

Table S3. ^{13}C chemical shifts of mobile molecules. All components are identified from ^{13}C DP-based INADEQUATE spectra. Superscripts are used to denote different allomorphs. Weak signals or minor species are indicated using "w" Not applicable (/). Unidentified (-).

DP-INADEQUATE	Type	C1	C2	C3	C4	C5	C6	OMe
Arabinose	A	105.1	82.7	76.9	82.1	62.1	/	/
		104.5	-	-	-	-	/	/
GalA Galacturonic acid	GalA	98.8	68.3	70.3	76.5	-	-	/
Glucose	Glc	92.8	71.8	73.2	70.0	73.0	61.1	/
		96.6	75.0	-	-			/
		-	-	-	-	70.0	64.0	/
		-	-	-	70.4	76.5	61.5	/
		-	-	-	-	72.5	64.7	/
		-	-	-	-	72.1	61.5	/
Xylan	Xn	104.1	77.4	75.0	81.9	63.3	/	/
		102.3	76.5	75.3	81.2	63.1	/	/
Lignin	S_a	140.44 (w)	103.9	154.1	134.9	154.1	103.9	56.1
	S_b	-	106.7	154.3	137.5	154.3	106.7	56.1
	G	-	-	149.6	149.6	-	-	-
	FA	-	-	149.6	149.6	113.71	-	-

Table S4. The crystalline structure of fibril (n=3). Values are presented as mean±s.e.

**Crystalline plane**	**1–10**	**110**	**200**
Peak position ($Å^{-1}$)	1.04±0.01	1.15±0.00	1.55±0.00
Scherrer width (L, Å)	31.6±0.18	31.6±0.18	31.6±0.18
d-spacing (d, Å)	6.0±0.03	5.5±0.01	4.1±0.00

Table S5. The dipolar order parameter S_{CH}. ^{13}C-^{1}H dipolar coupling and dipolar order parameters of biopolymers for ^{13}C CP and quantitative DP. Unidentified (-). Error estimates are based on the NMR signal-to-noise ratio.

Site	Dip Coupling, CP	S_{CH}, CP	Dip Coupling, DP 40s	S_{CH}, DP 40s
i4	12.5±0.16	0.95	12.0±0.26	0.92
s4	12.5±0.10	0.95	12.2±0.14	0.93
i/s/^{2f} Xn	12.1±0.05	0.92	11.7±0.07	0.89
C2	12.3±0.04	0.94	10.8±0.05	0.82
C3	12.3±0.04	0.94	11.8±0.04	0.9
^{3f}Xn1	12.1±0.14	0.92	9.7±0.16	0.74
^{2f}Xn4	11.8±0.11	0.90	9.5±0.21	0.73
^{3f}Xn4	12.4±0.03	0.95	10.4±0.05	0.79
R4	-	-	4.7±0.20	0.33
R3	-	-	5.0±0.10	0.35
Rgc1	-	-	7.9±0.79	0.55
Stb1	-	-	8.7±1.30	0.61

Table S6. ^{1}H-$T_{1\rho}$ relaxation times of lignin and polysaccharides. The data are fit using a single exponential equation: $I(t) = e^{-t/T}$. Standard errors of the fitting parameters are used as error bars.

Site	Atom (ppm)	T1, CP (ms)
FA9	169.9	14.8±0.8
G/FA 3/4	149.6	7.9±1.9
S2/6	103.9	16.1±0.8
OMe	56.3	11.7±0.2
i/s/^{2f}Xn1	105	22.2±0.7
i4	88.6	31.8±2.9
s4	83.7	20.7±0.7
i6	64.6	19.8±0.4
s6	62.4	15.5±0.3
AcCo	173.5	4.8±0.5
Ara (a) 1	107.8	13.9±0.8
Ara (b) 1	109.7	14.4±1.1
^{3f}Xn1	102.5	7.3±0.6
^{2f}Xn4	81.8	12.1±0.4
^{3f}Xn4	77.7	8.7±0.5
^{2f,3f}Xn5	63	3±0.3
AcMe	20.9	7.4±0.4
Rgc1	98.6	1.4±0.1
Rg3	69.9	5.9±0.4
R5	68.7	4.7±0.3

Table S7. ^{13}C-T_1 relaxation times of lignin and polysaccharides. The data are fit using single exponential equations $I(t) = 1{-}2e^{-t/T}$ for T1, DP (inversion recovery), and $I(t) = e^{-t/T}$ for T1, CP (Torchia CP). Standard errors of the fitting parameters are used as error bars. Unidentified (-).

Type	Atom (ppm)	T1, CP (s)	Atom (ppm)	T1, DP (s)
FA9	169.9	3.9±0.6	169.9	5±0.4
S3/5	153	8.2±0.7	154.5	4.9±0.5
G/FA 3/4	-	-	148.5	4.9±0.5
S4	-	-	135	6.4±0.7
S2/6	103.9	4.0±0.5	103.8	6±0.5
OMe	56.1	8.7±0.3	56.4	4.6±0.5
i/s/^{2f}Xn1	105	5.8±0.3	105	6.2±0.5
i4	88.6	6.9±0.1	88.6	7.1±0.6
s4	84	5.9±0.1	83.6	5.4±0.5
i6	65	5.5±0.4	65	3.3±0.5
s6	62.5	4.5±0.4	62.7	3.2±0.5
AcMe	-	-	21	5.2±0.5
AcCo	-	-	173	4.9±0.5
Ara (a) 1	-	-	107.9	5.2±0.6
Ara (b) 1	-	-	109.7	5.4±0.8
^{3f}Xn1	102.5	3.5±0.3	102.4	2.3±0.3
^{2f}Xn4	82	4.9±0.4	82.6	3.6±0.5
^{3f}Xn4	77.7	5.5±0.3	77	3.3±0.4
^{2f}Xn5	64	4.4±0.4	64	1.6±0.3
^{3f}Xn5	63	4±0.4	63	2.5±0.4
Rgc1	-	-	98.7	0.9±0.1
Sta1	-	-	92.8	0.5±0.1
Stb1	-	-	96.7	0.5±0.1
Rg3	-	-	70	0.8±0.1
R5	-	-	68.4	0.8±0.1

Table S8. Water-edited intensities of biopolymers. The intensity ratio is derived by comparing water-edited and control 2D correlation spectra.

Type	Carbon site	Raletive Intensity (S/S0)	Average value
Interior cellulose	^{a}i4-1	0.29	0.24±0.08
	^{a}i4-3	0.32	
	^{a}i4-2/5	0.30	
	^{b}i4-1	0.29	
	^{b}i4-3	0.13	
	^{b}i4-2/5	0.35	
	^{c}i4-1	0.20	
	^{c}i4-3	0.13	
	^{c}i4-2/5	0.35	
	^{d}i4-1	0.19	
	^{d}i4-3	0.29	
	^{d}i4-2/5	0.17	
Surface cellulose	^{e,h}s4-1	0.38	0.36±0.06
	^{e,h}s4-3	0.31	
	^{e,h}s4-2/5	0.28	
	^{f}s4-1	0.45	
	^{f}s4-3	0.32	
	^{f}s4-2/5	0.29	
	^{g}s4-1	0.47	
	^{g}s4-3	0.36	
Two-fold xylan	Xn^{2f}4-3	0.35	0.40±0.13
	Xn^{2f}4-2	0.43	
	Xn^{2f}4-1	0.42	
	Xn^{2f}3-5	0.43	
	Xn^{2f}5-1	0.22	
	Xn^{2f}5-4	0.27	
Mixed	$Xn^{3f,2f}$2/3-1	0.31	0.34±0.02
	$Xn^{3f,2f}$2/3-4	0.36	
	$Xn^{3f,2f}$2/3-5	0.33	
Three-fold xylan	Xn^{3f}5-1	0.53	0.40±0.08
	Xn^{3f}5-4	0.29	
	Xn^{3f}4-1	0.42	
	Xn^{3f}4-2	0.34	
	Xn^{3f}4-3	0.45	
	Xn^{3f}4-5	0.37	

Table S9. Intermolecular interactions of polymers. In total, 97 restraints are identified, including 36 strong interactions, 28 medium ones, and 33 weak interactions.

Interaction type	Strong interactions	Medium interactions	Weak interactions	Total
Cellulose-Xylan Ac	1	0	2	3 (3.1%)
Lignin-Cellulose	4	3	9	16 (16.5%)
Lignin-Lignin	8	11	6	25 (25.5%)
Lignin-Xylan	11	13	15	39 (40.2%)
Lignin-Mixed sugar	12	1	1	14 (14.4%)
Sum	36	28	33	97 (100%)

Percentage of the interaction site

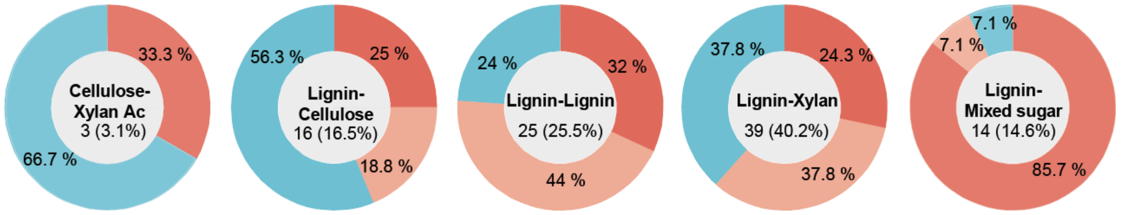

References

(1) Van Erven, G.; De Visser, R.; Merkx, D. W. H.; Strolenberg, W.; De Gijsel, P.; Gruppen, H.; Kabel, M. A. Quantification of Lignin and Its Structural Features in Plant Biomass Using[13] C Lignin as Internal Standard for Pyrolysis-GC-SIM-MS. *Anal. Chem.* **2017**, *89* (20), 10907–10916. https://doi.org/10.1021/acs.analchem.7b02632.

(2) Nishiyama, Y. Molecular Interactions in Nanocellulose Assembly. *Philos. Trans. R. Soc. A: Math. Phys. Eng. Sci.* **2017**, *376* (2112), 20170047. https://doi.org/10.1098/rsta.2017.0047.

(3) Wen, J.; Sun, S.; Xue, B.; Sun, R. Quantitative Structural Characterization of the Lignins from the Stem and Pith of Bamboo (*Phyllostachys Pubescens*). *Holzforschung* **2013**, *67* (6), 613–627. https://doi.org/10.1515/hf-2012-0162.

(4) Kirui, A.; Zhao, W.; Deligey, F.; Yang, H.; Mentink-Vigier, F.; Wang, T. Carbohydrate-Aromatic Interface and Molecular Architecture of Lignocellulose. *Nat. Commun.* **2022**, *13* (1), 538. https://doi.org/10.1038/s41467-022-28165-3.

(5) Kang, X.; Kirui, A.; Dickwella Widanage, M. C.; Mentink-Vigier, F.; Cosgrove, D. J.; Wang, T. Lignin-Polysaccharide Interactions in Plant Secondary Cell Walls Revealed by Solid-State NMR. *Nat. Commun.* **2019**, *10* (1), 347. https://doi.org/10.1038/s41467-018-08252-0.

(6) Simmons, T. J.; Mortimer, J. C.; Bernardinelli, O. D.; Pöppler, A. C.; Brown, S. P.; DeAzevedo, E. R.; Dupree, R.; Dupree, P. Folding of Xylan onto Cellulose Fibrils in Plant Cell Walls Revealed by Solid-State NMR. *Nature Communications* **2016**, *7*, 1–9. https://doi.org/10.1038/ncomms13902.

(7) Kang, X.; Zhao, W.; Dickwella Widanage, M. C.; Kirui, A.; Ozdenvar, U.; Wang, T. CCMRD: A Solid-State NMR Database for Complex Carbohydrates. *J. Biomol. NMR* **2020**, *74* (4–5), 239–245. https://doi.org/10.1007/s10858-020-00304-2.
